# A microscale soft ionic power source modulates neuronal network activity

**DOI:** 10.1038/s41586-023-06295-y

**Published:** 2023-08-30

**Authors:** Yujia Zhang, Jorin Riexinger, Xingyun Yang, Ellina Mikhailova, Yongcheng Jin, Linna Zhou, Hagan Bayley

**Affiliations:** 1 Department of Chemistry, University of Oxford, Oxford, UK; 2 Ludwig Institute for Cancer Research, Nuffield Department of Medicine, University of Oxford, Oxford, UK

**Keywords:** Biomedical engineering, Energy, Biomimetics

## Abstract

Bio-integrated devices need power sources to operate^1,2^. Despite widely used technologies that can provide power to large-scale targets, such as wired energy supplies from batteries or wireless energy transduction^3^, a need to efficiently stimulate cells and tissues on the microscale is still pressing. The ideal miniaturized power source should be biocompatible, mechanically flexible and able to generate an ionic current for biological stimulation, instead of using electron flow as in conventional electronic devices^4–6^. One approach is to use soft power sources inspired by the electrical eel^7,8^; however, power sources that combine the required capabilities have not yet been produced, because it is challenging to obtain miniaturized units that both conserve contained energy before usage and are easily triggered to produce an energy output. Here we develop a miniaturized soft power source by depositing lipid-supported networks of nanolitre hydrogel droplets that use internal ion gradients to generate energy. Compared to the original eel-inspired design^7^, our approach can shrink the volume of a power unit by more than 10^5^-fold and it can store energy for longer than 24 h, enabling operation on-demand with a 680-fold greater power density of about 1,300 W m^−3^. Our droplet device can serve as a biocompatible and biological ionic current source to modulate neuronal network activity in three-dimensional neural microtissues and in ex vivo mouse brain slices. Ultimately, our soft microscale ionotronic device might be integrated into living organisms.

## Abstract (web summary)

A study describes the development of a miniaturized hydrogel-based soft power source capable of modulating the activity of networks of neuronal cells without the need for metal electrodes.

## Main

Soft microscale power sources promise increased biocompatibility and flexibility compared to conventional bulky batteries^9^. The electric organ of the electric eel is an example of a biological energy source, which uses ion fluxes to generate electricity. Although a few studies have investigated the electrogenic behaviour of the organ and others have developed large power arrays over hundreds of square centimetres that mimic this behaviour^7,8^, none has created multicompartment microscale ionic power sources that can be turned on on-demand, and interact with living cells.

Here we report a miniaturized soft power source made by depositing nanolitre lipid-supported hydrogel droplet networks^10–12^ that use internal ion gradients to generate energy output. The droplet power source stores energy at high density and is biocompatible, mechanically flexible, scalable and portable after encapsulation. We demonstrate that the attachment of neuron-containing droplets with the droplet device enables ionic current modulation of neuronal network activity by stimulating intracellular Ca^2+^ waves.

## A bioinspired soft ionic power source

**Fig. 1:**
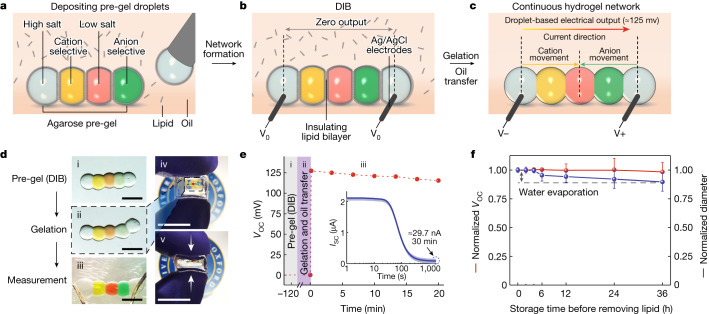
Structure and output performance of the droplet power source. **a**–**c**, Fabrication process for a power unit formed by depositing hydrogel droplets: pre-gel droplets were submerged in lipid-containing oil and acquired lipid monolayer coatings, which subsequently formed lipid bilayers when droplets were placed in contact (**a**); the insulating lipid prevented ion flux between droplets when they were connected to form a single unit (**b**); Ag/AgCl electrodes were used to measure electrical output, and the power source was activated by transfer into lipid-free oil and thermal gelation to rupture the lipid bilayers (Methods; **c**). The current direction within the device is from left to right; that is, cations move from the left to the central droplet and anions move from the right to the central droplet. **d**, Bright-field images of the formation process of a droplet power unit. In (i) to (iii), the volume of each droplet was 50 nl. Scale bars, 500 μm. Panel (iii) shows the insertion of Ag/AgCl electrodes. In (iv) and (v), droplets were encapsulated in a flexible and compressible organogel to demonstrate energy preservation in a portable unit. The volume of each droplet was 500 nl. Scale bars, 10 mm. **e**, Output open-circuit voltage (*V*_OC_) during the transition from pre-gel (i) to gel (ii) to continuous hydrogel network (iii), as shown in **d**. Inset: output short-circuit current (*I*_SC_) of a droplet power unit after formation of a continuous hydrogel network (iii). **f**, Variation of normalized *V*_OC_ and mean droplet diameter of single power units with different length of storage time in oil before the formation of continuous hydrogel networks. Normalization was with respect to the initial values of each experiment. The diameter of the droplets decreased over time owing to the evaporation of water. Data in **e**,**f** are mean values ± s.d. (*n* = 7). Source data

**Extended Data Fig. 1:**
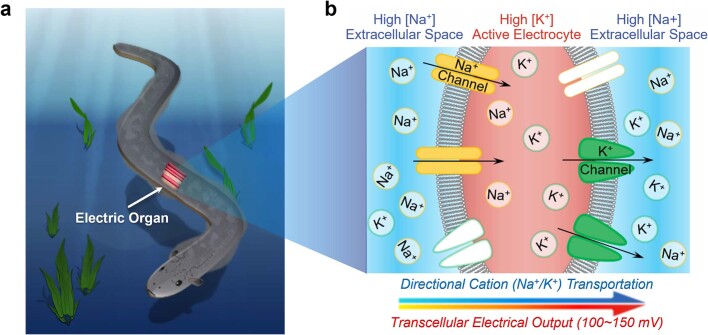
Electricity-generating mechanism of the electric eel. **a**, Location of the electric organ in the electric eel (Electrophorus electricus). **b**, Electrocytes stacked within the organ generate an electrical potential, which arises as depicted from directional ion fluxes through sodium and potassium channels.

**Extended Data Fig. 2:**
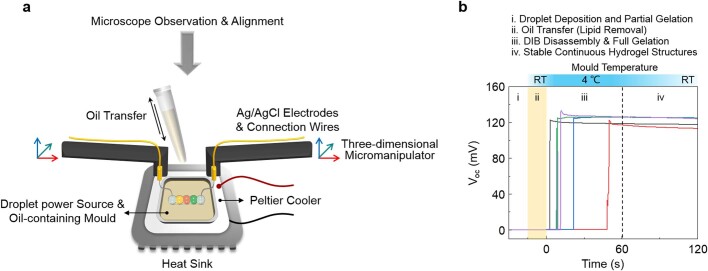
Measurement of power source activation during DIB rupture. **a**, The integration of a Peltier cooler and a heat sink to the bottom of the droplet measurement system enabled electrical readouts during temperature-triggered droplet gelation and oil transfer. **b**, Output V_OC_ during droplet network formation (i) and the activation process (ii and iii). Five independent droplet power sources were all activated within 60 s (iv).

The electricity-generating capability of the electric eel (for example, *Electrophorus electricus*) relies on stacking thousands of electrocytes in series (Extended Data Fig. 1), in which the cations Na^+^ and K^+^ can pass unidirectionally through ion-selective protein channels in the cell membranes driven by concentration gradients^13,14^. We mimicked the general layout and mechanism of the eel’s electric organ by combining five aqueous nanolitre pre-gel (agarose) droplets in sequence (Fig. 1a). In a single unit, the droplets were in the order: high-salt (for example, CaCl_2_, KCl or NaCl), cation-selective, low-salt, anion-selective and another high-salt droplet. They were deposited in a lipid-containing oil by using an electronic microinjector (Methods). The droplets were initially surrounded by monolayers of lipid, which formed droplet interface bilayers (DIBs) within seconds following contact with one another, thereby creating a stabilized, support-free structure^12,15^ (Fig. 1b). To activate the power source, the assembled droplets were moved into lipid-free oil to remove the lipids and disassemble the DIBs. The droplets were then gelled at 4 °C to create a continuous hydrogel structure (Fig. 1c, Supplementary Fig. 1 and Extended Data Fig. 2).

A major advantage of our strategy is that, before transfer to lipid-free oil and gelation, each droplet is separated from its neighbours by lipid bilayers, which prevent ion flow between the droplets while mechanically stabilizing the structure. After disruption of the insulating lipid bilayers, ions moved through the conductive hydrogel, from high-salt to low-salt droplets, passing through the selectively permeable compartments^12,16^ (Supplementary Note 1). By using chemically active Ag/AgCl electrodes^10,17^, the energy released from the salt gradients was transformed into electricity, and the hydrogel structure could act as an energy source and power external components (Fig. 1d). The lipid plays a critical role, enabling the formation of a stable droplet network without energy dissipation and the on-demand activation of powering activity; our approach provides a means to build a soft ionic power source on a microscale, which has not been achieved previously^7,8^ to our knowledge (Supplementary Note 2).

**Extended Data Fig. 3:**
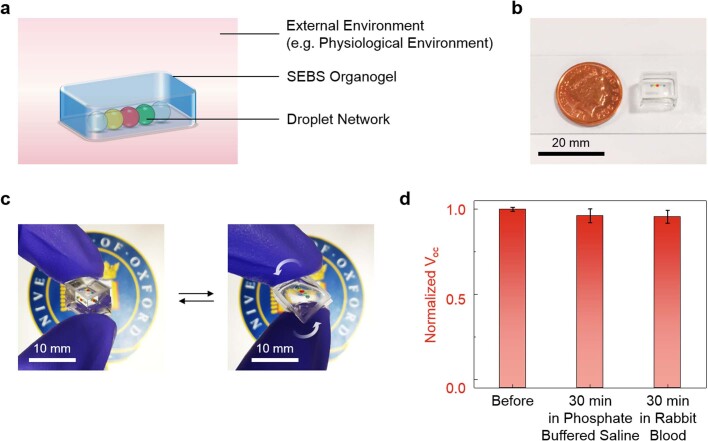
SEBS organogel encapsulation enabled preservation of droplet networks in physiological environments. **a**, Schematic showing the SEBS organogel encapsulation. **b**, Photographs of a droplet power source formed from 500 nL droplets and encapsulated in an organogel cube. Comparison with a one-penny coin (20.3 mm diameter). **c**, The encapsulated droplet power source is soft and can withstand repeated bending and twisting by hand. **d**, The open-circuit voltages are shown for droplet power sources encapsulated in organogel after 30 min immersion in phosphate buffered saline (pH 7.4, Gibco) or rabbit blood (New Zealand white rabbit strain, Envigo).

In addition to liquid oil, we also used a thermoreversible organogel^18,19^ to disassemble the DIBs and thereby create a freestanding, portable droplet power source. The organogel precursor poly(styrene-*b*-ethylene-*co*-butylene-*b*-styrene) triblock copolymer (SEBS) was dissolved in a high-melting-point alkane mixture to produce a gel–liquid transition temperature of below 37 °C (Supplementary Fig. 2). The molten organogel (37–40 °C) served as a lipid-free medium to replace the lipid-free oil used during DIB disassembly. The organogel solidified along with the droplet power source during the gelation process at 4 °C. After gelation, the organogel–droplet composite was gently detached from the mould to yield a freestanding, encapsulated droplet power source. The organogel encapsulation could support compression and twisting (Fig. 1d) and prevent ionic leakage in physiological environments (Extended Data Fig. 3), which greatly expands the portability of the droplet power source for potential implantable and wearable applications^20,21^.

The electrical outputs of a single power unit before and after activation were measured (Fig. 1e). After assembly, the droplets (50 nl each, 200-fold gradient in salt concentration) in a lipid-containing oil adhered to each other through the DIBs. We left the structures at ambient temperature (25 °C) for 5 min to allow the droplets to partially gel and reach their equilibrium contact angles. When we inserted electrodes into the two end droplets of high salinity, no current was recorded, indicating that the insulation of the DIBs prevented energy dissipation. Then, we removed the lipid with lipid-free oil and triggered full gelation at 4 °C for 1 min, thereby establishing an ionically conductive pathway. The activated droplet power source generated 127 mV at open circuit (*V*_OC_), a value comparable to the potential generated by a single electrocyte (100–150 mV). The short-circuit current (*I*_SC_) reached a peak value of 2.2 μA within seconds and then decreased to lower values owing to the limited quantities of the contained salts (100 nmol in each of the two high-salt droplets), in agreement with simulations (Supplementary Fig. 3). An output current persisted after 30 min with a value of about 30 nA. In addition, the droplet power source could be stored and used on-demand, as ensured by the robust hydrogel compositions (Supplementary Fig. 4) and insulating DIBs. We stored the droplet power sources in a lipid-containing oil to test energy preservation over time (Fig. 1f). After activation, the droplet power sources gave a less than 10% variation in the *V*_OC_ after 36 h storage. Over the same period, the volume of the droplets slightly decreased, owing to water loss.

## Output optimization

**Extended Data Fig. 4:**
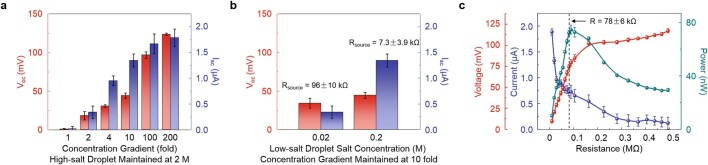
Optimization of the droplet power source for maximal output. **a**, V_OC_ and I_SC_ of single power units with different concentration gradients between the high-salt and low-salt droplets, with the high-salt droplets set at 2 M CaCl_2_. **b**, V_OC_ and I_SC_ of single power units with different salt concentrations in the low-salt droplets, with the concentration gradient set at 10-fold (see Supplementary Note 3 for detailed discussion). **c**, The dependence of output voltage, current, and power of a single power unit on the external loading resistance. The instantaneous output power reached a maximum (75 nW for 50 nL droplets) when the loading resistance was set around 78 kΩ. The output voltage, current, and power were typical of a concentration cell, showing a positive correlation between voltage and load resistance, while the current followed a reverse trend^17^. Data are presented as mean ± s.d. (n = 5).

To improve the output performance of the droplet power source, we analysed several key parameters that affect the electrogenic behaviour on the basis of the principle of reverse electrodialysis^17,22^: an ionic gradient across a selectively permeable membrane gives rise to an electromotive force across that membrane. First, the type of salt, concentration gradient and external resistance were optimized. Calcium chloride produced the highest output voltage compared to sodium chloride and potassium chloride at the same salt concentration (Supplementary Fig. 5a). Charged organic compounds can also create a concentration gradient under certain conditions and be used to build power sources (Supplementary Fig. 5b). Increasing the concentration ratio (gradient) between the high- and low-salt droplets increased the output voltage (Extended Data Fig. 4), but decreasing the low-salt concentration to produce a larger ratio resulted in a decrease in output current due to the increased internal resistance of the droplet power source (Supplementary Note 3). Then, using the optimal 200-fold CaCl_2_ gradient, the dependences of the output voltage, current and power on external resistance were measured, showing the resistance of a power unit made of 50-nl droplets to be about 78 kΩ and the maximum output power to be about 75 nW.

**Fig. 2:**
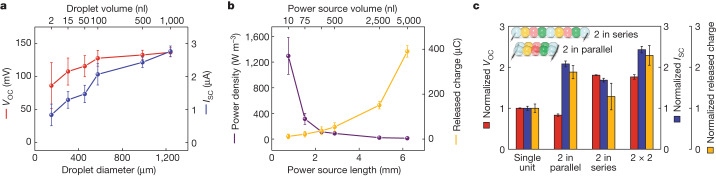
Effect of droplet volume on the electrical characteristics of droplet power sources. **a**, Initial (*t* = 0) *V*_OC_ and *I*_SC_ values. Droplet volumes below 100 nl were calculated on the basis of diameters measured by microscopy. **b**, Calculated power densities and total released charge of single power units with various droplet volumes. The power source volume and length were five times that of a single-droplet volume and diameter. **c**, Normalized *V*_OC_, *I*_SC_ and total released charge of power units formed into droplet networks in series and/or in parallel. The volume of each droplet was 1.84 nl. 2 × 2 stands for two sets of two paralleled power units in series. Inset: schematics showing the power units that were formed into continuous droplet networks during measurements. The normalization was with respect to the outputs of a single unit. Data are mean values ± s.d. (*n* = 5). Source data

One of the advantages of using lipid-supported hydrogel droplets to build soft power sources is the ease of miniaturization. Decreasing the volume of the droplets by 99.8% from 1,000 to 1.84 nl resulted in a concomitant decrease in output voltage (36%, from 136 to 87 mV) and current (70%, from 2.7 to 0.83 μA; Fig. 2a). These decreases may be attributed to an increase in the internal resistance of the droplets (Supplementary Note 3) and the increased concentration polarization^23^ across the selective droplets. However, the decreases were small compared to the decrease in volume; in fact, the average energy density at the matching resistance greatly increased for droplets of 1.84 nl by around 100 times to about 1,300 W m^−3^, representing an approximately 680-fold increase over the previous eel-inspired design^7^ and an approximately 5-fold increase over the subsequent paper–gel design^8^ (Fig. 2b and Extended Data Table 1). Although the total released charge was lower for the miniaturized power sources (Fig. 2b and Supplementary Fig. 6), we could combine multiple power units in series and/or in parallel to increase the output voltage and/or current. *V*_OC_ increases with the number of units in series; *I*_SC_ and the total released charge increase with the number of units in parallel (Fig. 2c).

## Scalable power source networks

**Fig. 3:**
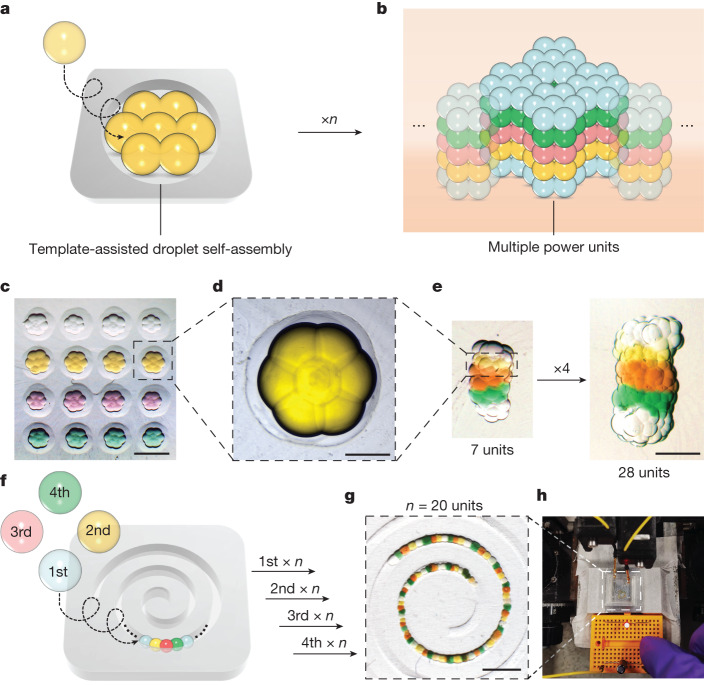
Template-assisted droplet network fabrication and output. **a**,**b**, Preparation of a large-scale patterned power source network. First, seven droplets were deposited in a mould, by using a programmable microinjector, and formed a hexagonal ‘flower-like’ structure (**a**). Droplet networks can be drawn into a truncated pipette tip by capillary action and arranged in three dimensions. Hexagonal assemblies of droplets were layered to form larger droplet networks (**b**). *n* refers to the number of units. **c**, Bright-field images of a mould with multiple droplet hexagons. The volume of each droplet was about 4 nl. Scale bar, 600 μm. **d**, Zoom-in of a single hexagonal layer. Scale bar, 200 μm. **e**, Stacks of 7 and 28 power units. Scale bar, 600 μm. **f**, After four-step sequential deposition into a spiral mould, droplets self-assembled into a chain of power units (Methods). **g**,**h**, Twenty power units were connected (**g**; scale bar, 1.2 mm) to generate an output voltage sufficient to light up a red light-emitting diode (**h**).

For larger-scale droplet networks, it is important to increase the contact area between different functional droplet layers without increasing the thickness of droplets. This is because the internal resistance of droplets is negatively correlated to the contact area and positively correlated to the thickness (Supplementary Note 3). Hence, keeping a low internal resistance—small size—while increasing the number of units in series or in parallel increases the output voltage or current, respectively. To scale up the assembly of small droplets for larger-scale applications, we adopted a template method, depositing multiple droplets into three-dimensionally printed resin moulds to produce power units of predesigned patterns (Fig. 3a,b). Template-assisted self-assembly of spherical units, ranging from the nanoscale to the microscale, has been widely used for fabricating patterned structures^10,24,25^; an attractive force between the units and confinement within the template are two conditions necessary for self-assembly^26^. In our approach, the formation of lipid-based DIBs provides an attractive force between droplets (spring constant of about 4 mN m^–1^, tensile strength of about 25 Pa)^11^, while the boundary of the mould limits their separation.

We fabricated cylindrical moulds with inner diameters of 600 μm, about three times larger than the diameter of a 4-nl droplet. In each mould, we deposited seven droplets (4 nl), which spontaneously assembled into a hexagonal ‘flower-like’ pattern within seconds (Fig. 3c,d and Methods). Next, we stacked five self-assembled droplet hexagons, with contents corresponding to the five droplets of a power unit, in a deeper cylindrical mould to form a larger power source network in three dimensions (Fig. 3e). An even larger network of 20 hexagons (28 units, 140 droplets) took less than 10 min to build (Fig. 3e). Automation of the construction process might be achieved with a three-dimensional droplet printer^11,27,28^ to produce droplet networks composed of thousands of power units.

To demonstrate the increased output of a multi-droplet assembly, we assembled 20 five-droplet units in series in a spiral mould (Fig. 3f). The high-salt droplets were deposited first, the cation-selective droplets second, the low-salt droplets third, and the anion-selective droplets last (Fig. 3g). The template confined the droplets during deposition, and the DIBs kept the droplets closely attached to each other in a chain. The structure was maintained after washing with lipid-free oil. The spiral power source could light up a light-emitting diode, which required an applied potential of about 2 V (Fig. 3h). The output power was sufficient for additional applications, such as charging a capacitor and powering a pulse generator (Supplementary Fig. 7). Notably, the droplet power sources could be recharged through electrodialysis by applying a reverse potential (200 mV) to the droplet network^29,30^, recovering greater than 60% of the original *I*_SC_ after ten discharges (Supplementary Fig. 8).

## Neuronal modulation by the generated ionic current

**Fig. 4:**
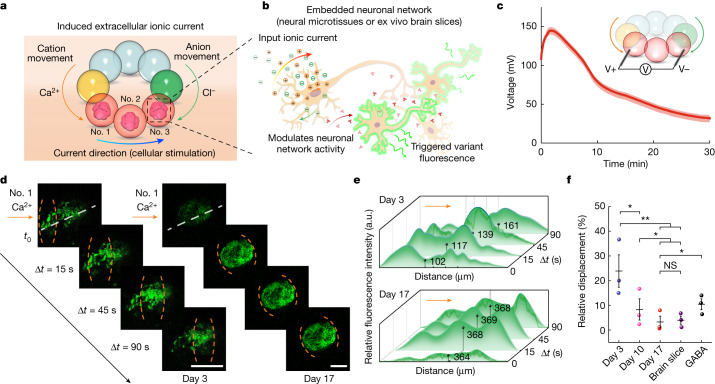
Neuronal modulation induced by the ionic droplet device. **a**, The triggering strategy used to modulate neuronal activity by generating ionic current from a droplet device. The high-salt and ion-selective droplets together acted as a droplet device, which was attached to droplets that contained neural microtissues or ex vivo mouse brain slices. Droplet no. 1, no. 2 and no. 3 received a cation influx from the left and an anion influx from the right. **b**, The ionic-current-modulated neuronal activity as reflected by intracellular Fluo-4 fluorescence. **c**, Output of the droplet device across the low-salt droplets. The voltage readout was conducted in open-circuit mode to ensure that the continuous hydrogel network was the only current path (*n* = 5). The volume of each droplet was 500 nl. The average voltage during the first 10 min was 120 mV. The corresponding ionic current was about 2.6 µA. **d**, Frames at various time points showing neurons embedded in droplet no. 1. Neurons were cultured for different periods (day 3 and 17), and reflected a change of neuronal network activities. The high-salt droplets contained 0.5 M CaCl_2_. Ionic current flowed from left to right into droplet no. 1. Orange dashed lines mark the modulated area. Scale bars, 150 μm. **e**, Relative fluorescence intensities at different time points along the white dashed lines indicated in **d**. The black dot in each plot indicates the centre of fluorescence (weighted-mean distance) at the corresponding time point. a.u., arbitrary units. **f**, Relative displacement of the centre of fluorescence over 90 s for neuronal networks after different culture periods and in ex vivo brain slices from mice. GABA treatment was used on the day-17 neural tissues to suppress activities of neuronal networks (*n* = 3; *P < 0.05; **P < 0.01; NS, not significant; unpaired one-tail t-test). Data in **f** are presented as mean values ± s.d. Source data

**Extended Data Fig. 5:**
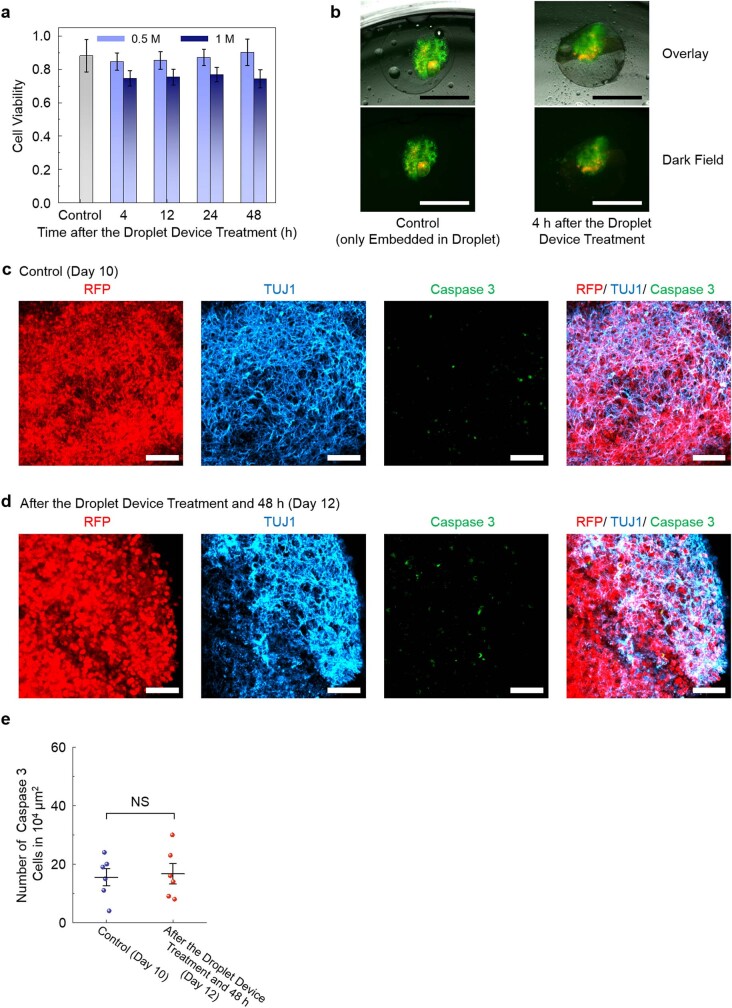
Cell viability study and immunofluorescence staining of neural microtissues before and after treatment of the droplet device. **a**, Cell viability in neuron-containing droplets, which had been connected to droplet devices for 10 min and then cultured in medium for 4, 12, 24, and 48 h. The control group was neural microtissues embedded in agarose droplets and not placed in contact with a droplet device. Salt concentrations in the high-salt droplets (CaCl_2_) were: 0.5 M (50-fold gradient, light blue); 1 M (100-fold gradient, dark blue). Data are presented as mean ± s.d. (n = 5). **b**, Fluorescence (bottom) and overlaid bright-field (top) live/dead imaging of microtissues embedded in agarose droplets. Calcein-AM (live, green) and PI (dead, red) staining were conducted after droplet device (0.5 M) modulation. Scale bars, 600 μm. **c** and **d**, RFP-labelled microtissues were stained for the neuronal cell marker TUJ1 and the apoptosis marker caspase 3. RFP is expressed by live cells; the TUJ1 staining reveals the neuronal cells including their processes; the caspase 3 staining reveals apoptotic cells. The control group (**c**, Day 10) was not contacted with a droplet device. The experimental group had been connected with the droplet device for 10 min and then cultured in medium for 48 h (**d**, Day 12). Salt concentrations in the high-salt droplets (CaCl_2_) were 0.5 M (50-fold gradient). **e**, The number of apoptotic cells in an area of 100 × 100 μm^2^. Data are presented as mean ± s.d. (n = 6).

We examined the influence of our droplet device on the activity of neurons. The high-salt and ion-selective droplets together can act as an open droplet device that can be attached to external components through its termini (Fig. 4a). When this open device is attached to droplets with lower ion concentrations, a conductive pathway is completed that allows the ionic current (about 2.6 µA) to flow through the attached droplets (Fig. 4b,c and Supplementary Fig. 9). If neurons are embedded in the low-salt droplets, the generated extracellular ionic current will modulate individual neuronal activity^4,31^ and, in turn, reflect neuronal network activities^32^. To test this, we used a microfluidic device to generate neural microtissues consisting of Matrigel spheres (about 570 μm  in diameter) laden with human neural progenitor cells. The neural microtissues were coated with low-salt agarose hydrogel containing neuron culture medium to form the neuron-containing droplets (Fig. 4a, red droplets; Methods and Supplementary Fig. 10). The droplet device was then attached to the neuron-containing droplets in a circular container (Supplementary Fig. 11). With 0.5 M CaCl_2_ in the high-salt droplets, the neurons retained high viability after 10 min of attachment as verified by cell viability assays with PrestoBlue, live–dead staining with Calcein AM and propidium iodide, and immunofluorescence staining with the neuronal marker TUJ1 and the apoptosis marker caspase 3 (Extended Data Fig. 5). Neuronal activities were measured by confocal imaging using Fluo-4 Direct as an intracellular calcium dye^28,33^, which does not respond to extracellular calcium (Methods, Supplementary Note 4 and Supplementary Fig. 12). Time-lapse recordings revealed the spatiotemporal course of neuronal modulation when the droplet device was attached to the neuron-containing droplets (Fig. 4d). The correlation of the neuronal activity with the ionic current indicated that the activity was caused by the droplet device and was not spontaneous^34,35^.

**Extended Data Fig. 6:**
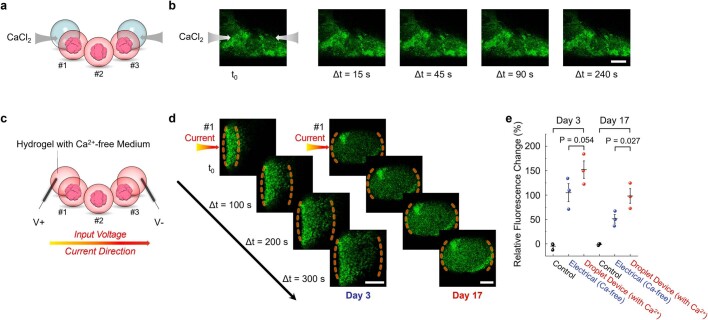
Droplet configurations for verification of the modulatory effects of droplet devices on neural microtissues. **a**, Direct contact of neuron-containing droplets (neurons cultured for 3 days) with high-salt droplets (0.5 M CaCl_2_). **b**, Frames at various time-points showing a low level of stable intrinsic fluorescence (amplified). Scale bar, 300 μm. **c**, Applying an external voltage to Ag/AgCl electrodes in Ca^2+^-free hydrogel droplets in contact with droplets containing neural microtissues. Droplet #1 received a positive input voltage relative to droplet #3. The resulting current was ~2.6 µA, which is similar to that produced by the droplet device. **d**, Frames at various time-points showing neurons embedded in the #1 droplet. The neurons had been cultured for different periods (3 and 17 days). Ionic current flowed from left to right into the #1 droplet. Red dashed lines mark the border of the modulated area of the Ca^2+^ wave. Scale bars, 150 μm. **e**, Relative fluorescence changes for neural microtissues after modulation by various means. The control group was neuron-containing droplets in direct contact with high-salt droplets (**a**). The “electrical” group was subjected to an external voltage source (**b**). The droplet device group is documented in Fig. 4.

**Extended Data Fig. 7:**
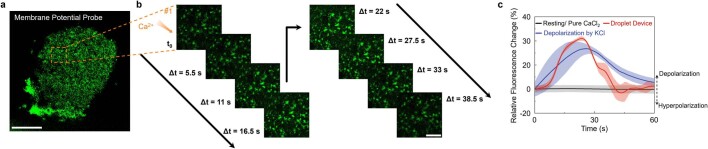
Monitoring the change of neuronal membrane potential after attachment of the droplet device. **a**, Neuronal membrane potential was measured by confocal imaging using FluoVolt™, a voltage-sensitive fluorescent probe (Methods). Scale bar, 200 μm. **b**, Frames at various time-points of a zoom-in area in (**a**) showing the fluorescence change after attachment to a droplet device. Ionic current flowed from top-left to bottom-right of the selected area. Scale bar, 50 μm. **c**, Relative fluorescence change of individual neurons after attachment of the droplet device. The black curve represents neurons in direct contact with high-salt droplets (0.5 M CaCl_2_). The stable fluorescence indicates that the neurons remain in a resting state. The red curve represents neurons under droplet device modulation (see **b**) and the blue curve represents neurons depolarized by adding 20 mM KCl solution. Three neural microtissues were tested under each condition and five cells from each were randomly selected for fluorescence quantification.

The modulation of neuronal activities was demonstrated by applying Ca^2+^-based ionic current to the neural microtissues after different culture periods (3, 10 and 17 days) and ex vivo mouse brain slices (Supplementary Fig. 13). The droplet device contained 0.5 M CaCl_2_ in the high-salt droplets. Direct contact with 0.5 M CaCl_2_ droplets did not produce ionic current and there was no significant intracellular calcium fluctuation in the neurons during 10 min of attachment (Extended Data Fig. 6). In comparison, when ionic current generated by the droplet device flowed through the neuronal network in droplet no. 1 from left to right, a stronger fluorescence was observed. Moreover, the intensified fluorescence spread directionally from left to right along the day-3 neural microtissue to form a wave-like fluorescence pattern^32^, indicating that the day-3 neural microtissues were locally modulated by the ionic current (Supplementary Video 1). To further verify that the modulation was induced by ionic current instead of by changing the extracellular concentration of specific ions (for example, Ca^2+^ and Cl^−^), we used Ag/AgCl electrodes to apply an electrical input to neural microtissues embedded in Ca^2+^-free droplets (Extended Data Fig. 6). As a result of the redox chemistry of the Ag/AgCl electrodes, the electrical input can induce an ionic current without creating a significant change in ion concentrations, for example, a Cl^−^ gradient. We observed a similar intracellular Ca^2+^ wave generated in the neuronal network by applying an input current intensity equivalent to that of our droplet device (Supplementary Note 5). Further, we applied a membrane-potential-sensitive dye, FluoVolt, to the neural microtissues during modulation with our droplet devices, and observed a depolarization of the  neuronal membrane potentials (Methods and Extended Data Fig. 7).

**Extended Data Fig. 8:**
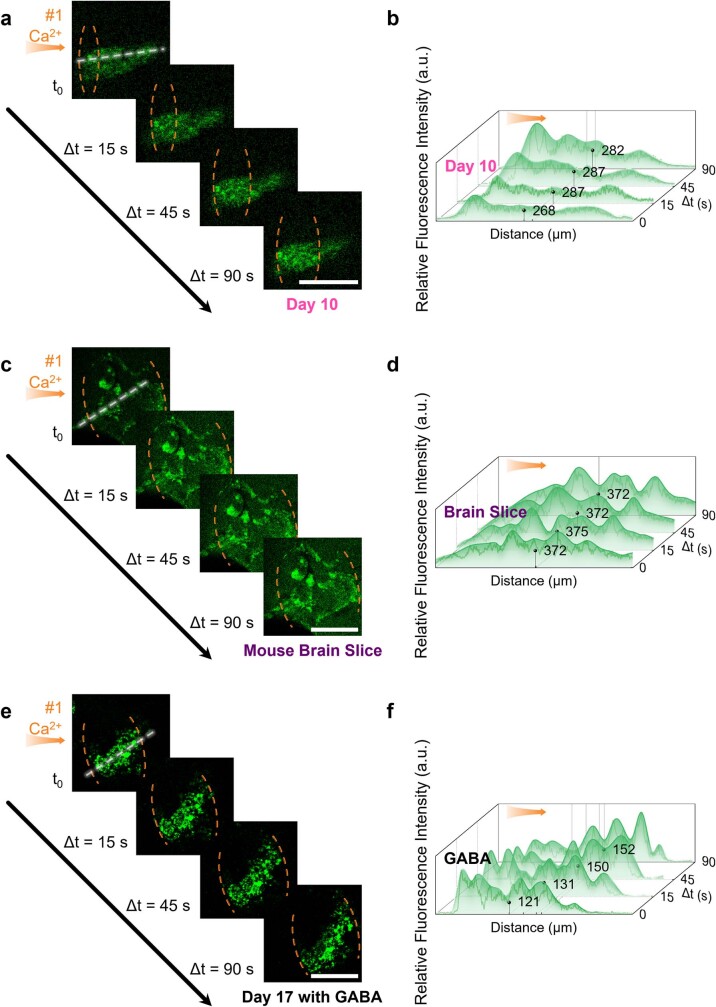
Effect of the droplet device on various neuronal networks. **a**, Neuronal network cultured for 10 days. High-salt droplets contained 0.5 M CaCl_2_. Ionic current flowed from left to right into the #1 droplet. Orange dashed lines mark the modulated area with increased fluorescence intensity. **b**, Profile plots of relative fluorescence intensities at different time-points along the white dashed line indicated in (**a**). Ionic current flowed from left to right. The black dot in each plot indicates the Weighted-mean Distance at that time-point. **c**, In the same setup, part of an *ex vivo* mouse brain slice was embedded in a hydrogel droplet for treatment with the droplet device. **d**, Profile plots for (**c**) and the Weighted-mean Distances at each time-point. **e**, In the same setup, day 17 neurons were treated with GABA before treatment with the droplet device. **f**, Profile plots for (**e**) and the Weighted-mean Distances at each time-point. Scale bars in (**a**), (**c**), and (**e**), 300 μm.

**Extended Data Fig. 9:**
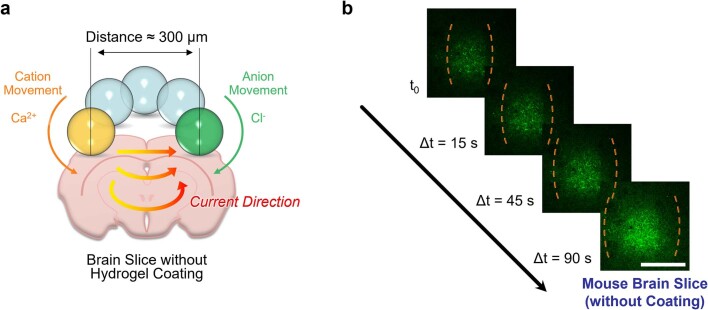
Direct attachment of the droplet device to a mouse brain slice. **a**, Direct response of a mouse brain slice without a hydrogel coating to the droplet device. High-salt droplets contained 0.5 M CaCl_2_. The two ion-selective droplets were separated by a distance of ~300 μm. **b**, Orange dashed lines mark the modulated area, which has increased fluorescence intensity. The fluorescence response of the brain slice was less directional and uneven compared to the neural microtissues, which might be due to the different neuronal wirings or tissue structures in different regions of the brain slice. Scale bar, 150 μm.

By contrast, in experiments using the day-17 neural microtissues, the neurons in droplet no. 1 were simultaneously modulated within 15 s in connection with the droplet device without showing the wave-like fluorescence pattern previously observed with the day-3 neural microtissues (Fig. 4d and Supplementary Video 2). We calculated the centre of fluorescence (weighted-mean distance) of each network to quantify the displacement of the ionic current modulation indicated by the fluorescence intensity (Fig. 4e and Methods). Day-17 neural microtissues showed similar fluorescence displacements to ex vivo mouse brain tissue, which were significantly lower than displacements in day-3 and day-10 neural microtissues (Fig. 4f and Extended Data Figs. 8 and 9). Day-17 neural microtissues also showed a faster propagating speed of the induced Ca^2+^ waves compared to day 3 counterparts, in agreement with a previous study^36^ (Supplementary Fig. 14). These results indicate that the neurons formed connected neuronal networks during prolonged culture and consequently showed simultaneous responses after the device attachment^37,38^. The ionic current from the droplet device presumably induced the release of excitatory messengers^32,39^, which resulted in simultaneous modulation throughout the interconnected neuronal network (Supplementary Note 5). To test our hypothesis that the presumed network activity was related to synaptic activity, we treated the day-17 neural microtissues with 30 μM γ-aminobutyric acid (GABA), which is an inhibitory neurotransmitter that lowers the intracellular potential and correspondingly the effectiveness of excitatory inputs^40^. The expression of GABA receptors in the neural progenitor cells was previously substantiated by immunofluorescence staining^41^. In the presence of GABA (Fig. 4f and Extended Data Fig. 8), neurons from the day-17 neural microtissues failed to exhibit simultaneous modulation; the wave-like fluorescence pattern was partially restored with increased fluorescence displacement under influence from the ionic current. These results indicate that the biocompatible droplet device can modulate neuronal network activities in neural microtissues.

## Outlook

The SEBS and other encapsulating methods may open the door to use the ionic power source for powering wearables and other mobile devices. There is however room for further improvement. The ideal power source should work in a physiological environment so it can be used in vivo for biological regulation. The present droplet power source uses temperature change to irreversibly trigger its activity and needs SEBS encapsulation to work in aqueous environments. A combination of aqueous transfer with the dewetting method^42^ and the use of light-controllable lipids^43^ or membrane proteins^44^ may achieve in vivo application by producing three-dimensionally printed droplet power sources in an aqueous environment with remote, reversible on-off switches. On this basis, incorporating other stimulus-responsive materials^12^, such as magnetic particles, into the hydrogel may endow remote-controlled mobility to perform in vivo energy delivery to confined biological environments. Future studies should focus on utilizing the device under physiological conditions and boosting the overall energy capacity, which could then be used to power next-generation bio-hybrid interfaces, implants, synthetic tissues and microrobots. The droplet device also paves an alternative path towards modulating the activity of various miniaturized cellular constructs, such as brain organoids^45,46^ and assembloids^47^.

## Methods

### Hydrogel materials

All materials were purchased from Sigma-Aldrich (Merck KGaA). For all droplet power sources, low-gelling-temperature (LGT) agarose was used to build the hydrogel scaffold. This material has enough gel strength for fabrication at around room temperature. Other materials were dissolved in Milli-Q water with 30 min ultrasonication (Branson 2800) and then mixed with the LGT agarose powder to form various precursor solutions (pre-gels). Final pre-gels had 2% w/v LGT agarose and the following compositions—high-salt hydrogel: 2 M CaCl_2_; low-salt hydrogel: 0.01 M CaCl_2_, 10% v/v poly(ethylene glycol) (number-average molecular weight 400). NaCl and KCl can replace CaCl_2_ if necessary (Supplementary Fig. 5). However, to obtain optimum output voltage, CaCl_2_ was used during electrical measurements except where noted. The cation-selective hydrogel contained 20% w/v poly(sodium 4-styrenesulfonate) (average molecular weight 70,000) and the anion-selective hydrogel contained 20% w/v poly(allylamine hydrochloride) (average molecular weight 50,000). Pre-gel solutions were first heated to 90 °C to dissolve agarose and then kept molten at 37 °C before and during droplet deposition. Food dyes were used only for photography and were absent during electrical recording and biological experiments.

### Preparing lipid–oil solutions

Agarose pre-gel droplets were deposited in a lipid-containing oil and acquired lipid coatings, which subsequently formed lipid bilayers at the interface (DIBs) when droplets were brought into contact. Lipids were purchased from Avanti Polar Lipids in powder form and stored at −80 °C. Undecane and silicone oil AR20 (Sigma-Aldrich) were filtered through 0.22-μm filters (Corning) under vacuum before use. Lipid films were prepared by bringing ampoules to room temperature and dissolving the lipids in anhydrous chloroform (Sigma-Aldrich) at 25 mg ml^−1^ to give the lipid stock solution. Using glass syringes (Hamilton), lipid stock solutions of 1,2-diphytanoyl-*sn*-glycero-3-phosphocholine (DPhPC, 90 μl) and 1-palmitoyl-2-oleoyl-*sn*-glycero-3-phosphocholine (POPC, 40 μl) were transferred into a Teflon-capped glass vial (Supelco, 7 ml) that had been cleaned with isopropanol. The chloroform was evaporated under a slow stream of nitrogen while the vial was rotated by hand to produce an even lipid film. The film was dried under vacuum for 24 h, and stored under nitrogen at −80 °C until use. When required for droplet fabrication, films were left at room temperate for 30 min, and then 2 ml of a pre-mixed solution of undecane and silicone oil (35:65 by volume) was added to the film, followed by sonication (Branson 2800) for 1 h. The total concentration of lipids was 2 mM with a molar ratio of DPhPC/POPC of 2:1. Lipid films were kept for a maximum of 2 months.

### Depositing droplet power sources

Droplets were formed in custom-made transparent resin mould, produced using a three-dimensional printer (Formlabs, Solid Print3D). Depending on the shape of the mould, various self-assembled patterns were formed. Typically, moulds were filled with 200 μl of lipid-containing oil. In each mould, droplets of pre-gel solution were deposited with a programmable microinjector (FemtoJet, Eppendorf), which ejected droplets from a loaded glass nozzle (Femtotips, Eppendorf) with volumes that ranged from femtolitres to microlitres. Single droplet power units were obtained by depositing droplets into contact with one another and allowing bilayers to form at the interfaces, which happened within seconds. Larger droplet networks self-assembled into predesigned shapes in templates, such as the hexagonal ‘flower-like’ pattern (Fig. 3). After formation, droplet networks, along with surrounding oil, were drawn into a truncated pipette tip by capillary action, and could then be rearranged; for example, stacked in in three dimensions by using micro-machined templates. An infrared radiation heater (Beurer, 150 W) was used to keep the temperature of the nozzles and the resin mould at approximately 37 °C. After fabrication, the droplet power sources could be stored for more than 2 days within a lipid–oil solution in a humid incubator at 37 °C to prevent water evaporation (Fig. 1f) without energy dissipation owing to the insulating DIBs.

### Triggering droplet power sources

To use a power source, the lipid insulation was removed by transferring the power source into oil without lipid and triggering full gelation at low temperature. To do so, the deposited droplet power sources were left for 5 min at ambient temperature (around 22 °C) to partially gel the agarose and allow the droplets to reach their equilibrium contact angles. Next, droplet power sources were washed with silicone oil by removing the lipid–oil solution from the mould and then adding 500 μl fresh silicone oil, containing no lipid. After the transfer to lipid-free oil, the droplet power sources were moved to a fridge (4 °C) for 1 min to allow complete disruption of the insulating DIBs and consequent formation of a continuous hydrogel structure. For in situ measurements of the electric output during the rupture of DIBs and low-temperature gelation, a Peltier cooler (14 W, 62 × 62 mm, RS PRO) and a heat sink (85 × 85 × 6 mm, RS PRO) were integrated to the bottom of the droplet measurement system (Extended Data Fig. 2). Such integration enabled the droplet deposition, power source activation and electrical measurement in an all-in-one setup.

### Encapsulating droplet power sources

A polymer-based organogel was prepared by mixing SEBS (molecular weight about 118,000, Sigma-Aldrich) with 1% by weight F68 flake (Pluronic, Sigma-Aldrich) and undecane–hexadecane oil (50:50 by volume) at a concentration of 20 mg ml^−1^. The mixture was then stirred at 95 °C in a closed vial. Once a clear liquid had been formed, it was cooled to 37–40 °C before use. Organogel encapsulation was conducted by replacing the silicone oil with the molten polymer–oil mixture at the last oil transfer step. Lipid-free organogel (1 ml) was used to wash and cover the droplet power sources. After the transfer to organogel, the encapsulated droplet power sources were moved to a fridge (4 °C) in which the organogel solidified. The final construct was gently extracted from the mould, forming a freestanding droplet power source. Electrodes can pierce through the solidified organogel for measurement of the power output.

### Characterization of droplet power sources

We used Ag/AgCl electrodes (100-μm diameter wire, Sigma-Aldrich) to contact the first and last compartments of the droplet power source, which were both high-salt droplets. The ion flux in the droplets was converted to electron flow in an external circuit (Supplementary Note 1). We recorded *V*_OC_ and *I*_SC_ using a Keithley 617 programmable multimeter set to voltage measurement mode with high input impedance (about 2 TΩ) or current measurement mode as a feedback-type picoammeter. The effective output power of the droplet power source was evaluated by monitoring the voltage and current with resistances ranging from 0.01 to 0.5 MΩ.

### Simulations

The output voltage and current of the droplet power source under various settings were simulated based on the experimental setup shown in Supplementary Fig. 3. We used COMSOL Multiphysics 5.6 and coupled Nernst Planck Poisson equations. The two ion-selective droplets were assumed to act as ion-exchange membranes with opposite fixed charges of 1,000 C m^−3^. The modelled ions were K^+^ and Cl^−^, with defined initial concentrations of 2 M and 0.01 M in the high-salt and low-salt droplets, respectively. Modelling conditions for interfaces were the combination of tertiary current distribution and the Nernst Planck interface, with Poisson-type charge conservation. Results were calculated using time-varied (transient) analysis with a time range from 0 to 1,800 s.

### Powering electronic components with droplet power sources

To light up a light-emitting diode (LED; Fig. 3h), the four types of droplets were deposited in a spiral mould (Fig. 3f) to form 20 power units in series connected to a red LED (Broadcom HLMP-K150). A capacitor (0.47 μF, RS PRO) could be connected in series to store the released energy from the droplet power source and subsequently light up the red LED (Supplementary Fig. 7a). A pulse generator circuit based on a 555-timer chip (TLC555IP, RS PRO) was also powered by the droplet power source (Supplementary Fig. 7c).

### Neuron culture and brain tissue collection

Neural progenitor cells (NPCs) were derived from human induced pluripotent stem cells (iPSCs), provided by Dr S. Cowley (James Martin Stem Cell Facility, Oxford). Neural differentiation of iPSCs and NPC culture were carried out according to published procedures^28,48^. NPCs were maintained as two-dimensional adherent cultures on Geltrex-coated (Life Technologies, A141133-02) culture plates in neural maintenance medium, which consists of N-2 medium and B-27 medium (1:1 v/v). The N-2 medium contains DMEM/F12 medium (Life Technologies; 21331020), 1× N-2 (Gibco, 17502048) and 1 mM GlutaMax (Gibco, 35050-038). The B-27 medium contains neurobasal medium (Gibco, 21103-049S), 1× B-27 (Gibco, 17504044) and 1 mM GlutaMax (Gibco, 35050-038). Day-26 (since neural induction) NPCs were collected by incubating with Accutase (Life Technologies, A11105-01) for 5 min at 37 °C and dissociated into a cell suspension with gentle pipetting. The cells were then centrifuged (5 min at 200*g*), and the supernatant was removed. Pre-thawed Matrigel (Corning) was added to the cell pellet and mixed to make a bio-ink with a cell density of 2 × 10^7^ cells per millilitre.

NPCs labelled with red fluorescent protein (RFP) were derived from RFP–iPSCs^28,48^. The cells were cultured and passaged in the same manner as the non-labelled NPCs except for the addition of 2.5 μg ml^−1^ puromycin (Thermo Fisher Scientific) in neural maintenance medium for RFP selection.

Mouse brain tissues were acquired from M. Lei. Adult C57BL/6 mice were killed following a Schedule 1 procedure. The brain was surgically removed, and 300-µm brain slices were prepared by using a Compresstome vibrating microtome (Precisionary, VF-300-0Z) equipped with an HP35-coated microtome blade (Thermo Fisher Scientific, 3150743). Brain slices were collected in chilled Earl’s balanced salt solution bubbled with carbogen (95% O_2_ and 5% CO_2_) and transferred onto 30-mm cell-culture inserts (Millicell, PICM0RG50) in six-well plates. The brain slices were incubated at 37 °C, with 5% CO_2_, for no more than 3 days in 75% BrainPhys medium with SM1 supplements (Stemcell Technologies, 05792), 25% horse serum (GIBCO, 16050130) and 100 U penicillin–streptomycin (GIBCO, 15140122).

### Fabrication of droplets containing cells or tissues

The procedure involved two major steps. First, we used a home-built microfluidic system to generate three-dimensional cellular microtissues^49^. Then, we cultured the microtissues and coated them with low-salt agarose hydrogel made from neuron culture medium, immediately before use to form a continuous hydrogel structure with an attached droplet power source (Fig. 4a).

In the first step of the construction of neural microtissues, the collected neural cells (NPCs) were pelleted and resuspended in Matrigel (Corning) and loaded into a syringe at 8 °C at 2 × 10^7^ cells per millilitre. The cell-laden Matrigel and oil (tetradecane, Sigma-Aldrich) were then pumped into a three-way polydimethylsiloxane (Sigma-Aldrich) connector by a programmable neMESYS syringe pump (Cetoni). At an optimized flow rate, spherical Matrigel droplets containing cells, separated by the carrier oil, were formed in a polytetrafluoroethylene tube (Cole-Parmer). The droplet diameter was determined by the inner diameter of the tube (for example, 570 μm). Then, the tube containing the cell-laden spherical microtissues and oil was placed in a culture chamber at 37 °C for 2 h to allow gelation of the Matrigel, thereby forming three-dimensional cell-laden microtissues. Finally, the microtissues were ejected from the exit tube, transferred to medium, and cultured before use. The day of forming neural microtissues was marked as day 0. The three-dimensional neural microtissues were cultured in a neural maintenance medium supplemented with 50 U ml^−1^ penicillin and streptomycin (Gibco, 15140-122). Cell medium was changed every 3 days^28^.

In the second step for embedding the neural tissues in agarose droplets, the cultured neural microtissues were transferred into a mould filled with silicone oil by using truncated pipette tips (200 μl). An infrared radiation heater was used to keep the surrounding temperature at approximately 37 °C. Residual medium was carefully removed before adding low-salt hydrogel solution (0.5 μl) with a 7000 series Hamilton syringe to coat each neural microtissue. The low-salt hydrogel contained 2% agarose, and about 1 mM Ca^2+^, about 4 mM K^+^ and about 140 mM Na^+^ from the neuron culture medium. Owing to the surrounding oil, the hydrogel solution rapidly covered the neural microtissues. Then, the mould was kept at 20 °C for 10 min to solidify the hydrogel coating. This final gel coating unified the size variation of different neural constructs, made them easy to handle, constrained the ionic current, and dissipated possible compressive forces on the embedded microtissues. The coated droplets were then returned to culture medium for dyeing and used for experiments on neuronal modulation. Ex vivo mouse brain slices could also be processed according to the same second step to obtain droplets containing this tissue.

### Neuron live–dead staining, viability determination and immunostaining

To image the live–dead distribution of neurons after power source modulation, neuron-containing droplets were incubated with 2.5 μM Calcein AM (C1430, Thermo Fisher Scientific) and 5.0 μM propidium iodide (Sigma-Aldrich) for 60 min at 37 °C before imaging with an epifluorescence microscope (Leica DMi8). PrestoBlue assays (Thermo Fisher Scientific) were used to determine live cell number and viability according to the manufacturer’s instructions. A microplate reader (CLARIOstar Plus) was used to quantify the fluorescence and hence the number of living cells.

For immunostaining, neural microtissues were first fixed in 4% v/v paraformaldehyde (Sigma-Aldrich) for 30 min at room temperature and then quenched in 50 mM glycine (Sigma-Aldrich). The samples were incubated with blocking solution, 5% donkey serum in Triton phosphate-buffered saline containing 0.1% v/v Triton X-100 (Thermo Fisher Scientific) for 1 h at room temperature. Primary antibodies to TUJ1 (Synaptic Systems) and caspase 3 (Thermo Fisher Scientific) were added in blocking solution and samples were incubated overnight at 4 °C. The next day, samples were washed three times (10 min each) in phosphate-buffered saline and then incubated with secondary antibodies for 2 h at room temperature. Samples were then washed in phosphate-buffered saline another three times (10 min each), followed by incubation with 4′,6-diamidino-2-phenylindole (5 μg ml^−1^) in Triton phosphate-buffered saline for 15 min and a final wash. *Z*-stack images of all immunostained neural microtissues were acquired using a fluorescence confocal microscope (Leica SP5).

### Neuronal modulation by droplet devices

A droplet device consisting of three high-salt and two ion-selective droplets was deposited in a circular container that was integrated upon an imaging dish (µ-Dish, Ibidi). Droplets formed a continuous hydrogel structure after oil transfer with lipid-free oil. The droplet device was then attached to three low-salt hydrogel droplets that contain neural microtissues or brain tissues, completing a ring structure (Supplementary Fig. 11). The droplet device could then produce ionic current that flows over neurons or tissues in the closed loop. Droplets containing neurons or brain tissues were combined with droplet devices for 10 min and then put back in culture medium for 20 min, as one modulation–relaxation cycle. Neurons recovered to the initial active state after each cycle (Extended Data Fig. 5). To investigate the network interaction with the droplet device, neuron-containing droplets were treated with GABA (Sigma-Aldrich) at a concentration of 30 μM, which has previously been determined to be an inhibitory but nontoxic concentration^40^.

### Neuronal imaging

For calcium imaging, a Fluo-4 Direct calcium assay kit (Invitrogen, F10471) was used according to the manufacturer’s instructions to measure calcium activity. Briefly, droplets containing neurons or brain tissues were transferred to 48-well plates and incubated with neural maintenance medium and Fluo-4 calcium imaging reagents (1:1 v/v) for 1 h at 37 °C. Time-lapse (XYZTime) fluorescence images were acquired at 1.28 s per frame under the optical settings suggested by Invitrogen by using a fluorescence confocal microscope (Leica SP5) at Ex/Em 488/525 nm. *Z*-stack images were acquired between the bottom of neural microtissues to about 50 μm above with a step of 5 μm per image. Maximum *Z* projection was then conducted to generate the final time-lapse images. Bright-field images were recorded with a stereomicroscope (Leica EZ4 W) and a wide-field light microscope (Leica DMi8). Images were processed using the Leica Application Suite X and Fiji (ImageJ).

For membrane potential imaging, a FluoVolt membrane potential kit (Thermo Fisher Scientific) was used according to the manufacturer’s instructions to measure neuronal membrane potential. Time-lapse fluorescence images were acquired at 0.37 s per frame under the optical settings suggested by the manufacturer by using a fluorescence confocal microscope (Leica SP5). Images were processed using the Leica Application Suite X and Fiji (ImageJ).

### Calculating imaging results

The fluorescence intensities of neurons were obtained using the Fiji freehand tool and profile plots function. To obtain the relative ion concentration distributions of Ca^2+^ and Cl^−^ on a selected line plot (Supplementary Fig. 9b), the relative concentration (*C*) is defined as

1 $$C=\frac{{\rm{Fluo}}(t)-{F}_{0}}{{F}_{{\rm{final}}}-{F}_{0}}$$

Fluo(*t*) is the fluorescence intensity at time *t*. *F*_0_ is the initial fluorescence intensity before forming a continuous hydrogel network. Owing to different fluorescence responses, *F*_final_ is the maximum fluorescence intensity across three droplets after 20 min for Ca^2+^ (fluorogenic response) and the minimum for Cl^−^ (quenching response).

To calculate the moving speed of Ca^2+^ waves across a neuronal network (Fig. 4d,e), we needed to first calculate the centre of fluorescence of the neuronal network. We chose the method of weighted mean to represent the position of the centre of fluorescence, which is defined as

2 $${\rm{Weighted-mean\; distance}}=\frac{\sum ({\rm{Intensity}}\times {\rm{Distance}})}{\sum {\rm{Intensity}}}$$

∑Intensity is the summation of each fluorescence value on a selected line plot. ∑(Intensity × Distance) is the summation of each fluorescence value multiplied by the distance from the origin (boundary of cells or brain tissues) of the selected line plot. Knowing the position of the centre of fluorescence, we can calculate the relative displacement of fluorescence (Fig. 4f), which is defined as

3 $${\rm{Relative\; displacement}}=\frac{\triangle ({\rm{Weighted\; -\; mean\; distance}})}{{\rm{Total\; length}}}$$

∆(Weighted-mean distance) is the variation of weighted-mean distance before and after attachment of the droplet device. Total length is the length of the selected line plot.

### Statistics

Statistical analyses were carried out using Origin and the *P* value was determined by unpaired one-way analysis of variance. Each experiment used a minimum of three independent droplet power sources.

### Reporting summary

Further information on research design is available in the Nature Portfolio Reporting Summary linked to this article.

## Online content

Any methods, additional references, Nature Portfolio reporting summaries, source data, extended data, supplementary information, acknowledgements, peer review information; details of author contributions and competing interests; and statements of data and code availability are available at 10.1038/s41586-023-06295-y.

### Supplementary information

Supplementary Information

Reporting Summary

Supplementary Video 1 Ionic current modulation based on day-3 neural microtissues. The ionic current generated by the droplet device flowed through the neuronal network from left to right. The intensified fluorescence spread directionally from left to right along the day-3 neural microtissue to form a wave-like fluorescence pattern.

Supplementary Video 2 Ionic current modulation based on day-17 neural microtissues. The ionic current generated by the droplet device flowed through the neuronal network from left to right. Neurons were modulated without showing the wave-like fluorescence pattern previously observed with the day-3 neural microtissues.

### Source data

Source Data Fig. 1

Source Data Fig. 2

Source Data Fig. 4

## Figures and Tables

**Extended Data Table 1:**
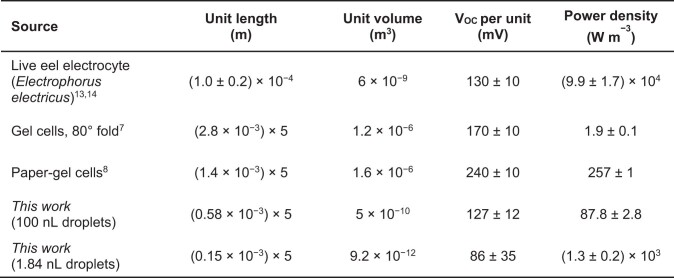
Comparison of geometrical and electrical characteristics of natural electrocytes, artificial electric organs, and droplet power sources of two different volumes

Where applicable, values are presented as mean ± s.d.

## Data Availability

All data generated during this study are included in the paper and its Supplementary Information. Data are also available from the corresponding authors on request. Source data are provided with this paper.
