# Supplementary material for: A microscale soft ionic power source modulates neuronal network activity
Source: Nature. 2023 Aug 30;620(7976):1001–6. doi: 10.1038/s41586-023-06295-y (PMC10468398; doi:10.1038/s41586-023-06295-y)
Supplement: Supplementary file 1 — Supplementary Information [file 41586_2023_6295_MOESM1_ESM.docx]

**Supplementary Information**

**A Microscale Soft Ionic Power Source Modulates Neuronal Network Activity**

Yujia Zhang^1✉^, Jorin Riexinger^1^, Xingyun Yang^1^, Ellina Mikhailova^1^, Yongcheng Jin^1^, Linna Zhou^1,2✉^ and Hagan Bayley^1✉^

^1^Department of Chemistry, University of Oxford, Oxford, UK.

^2^Ludwig Institute for Cancer Research, Nuffield Department of Medicine, University of Oxford, Oxford, UK.

^✉^e-mail: yujia.zhang@chem.ox.ac.uk;

linna.zhou@ludwig.ox.ac.uk;

hagan.bayley@chem.ox.ac.uk

Table of contents

Supplementary Note 1............................................................................................................ 2

Supplementary Note 2............................................................................................................ 4

Supplementary Note 3............................................................................................................ 5

Supplementary Note 4............................................................................................................ 7

Supplementary Note 5............................................................................................................ 7

Supplementary Fig. 1............................................................................................................. 9

Supplementary Fig. 2........................................................................................................... 10

Supplementary Fig. 3........................................................................................................... 11

Supplementary Fig. 4........................................................................................................... 12

Supplementary Fig. 5........................................................................................................... 13

Supplementary Fig. 6........................................................................................................... 14

Supplementary Fig. 7........................................................................................................... 15

Supplementary Fig. 8........................................................................................................... 16

Supplementary Fig. 9............................................................................................................17

Supplementary Fig. 10......................................................................................................... 18

Supplementary Fig. 11......................................................................................................... 19

Supplementary Fig. 12......................................................................................................... 20

Supplementary Fig. 13......................................................................................................... 21

Supplementary Fig. 14......................................................................................................... 22

Reference ............................................................................................................................23

**Supplementary Note 1. Theoretical background for the droplet power source**

In brief, the mechanisms of the ionic droplet power source can be described in four parts (Schematic S1).

**1.1 Part 1**

The direct current droplet power source is based on droplets containing different salt concentrations connected by charge-selective droplets that act as ion filters. Ions flow from high to low concentration through the charge-selective droplets, a process combining diffusion and Donnan exclusion, releasing the chemical energy stored in the ion concentration gradient (Schematic S1a) and giving rise to an electromotive force across the charge-selective droplets, as embodied in the Nernst-Planck equation and the Goldman-Hodgkin-Katz current equation^1,2^. Briefly, the flux of ions *s* in an electric field is given by $J_{S}$ (mol m^-2^ s^-1^), where:

$J_{S}=-D_{S}\left( \nabla C_{S}+\frac{FZ_{S}}{RT}C_{S}\nabla\varphi\right)$ (S1)

where $D_{S}$ (m^2^ s^-1^) is the diffusion coefficient of *s* in its medium, $C_{S}$ (mol m^-3^) is the molar concentration of *s*, $F$ (C mol^-1^) is Faraday’s constant, $Z_{S}$ is the charge of *s*, $R$ (J mol^-1^ K^-1^) is the gas constant, $T$ (K) is the temperature, and $\varphi$ (V) is the electrical potential. The ion flux in current form, using assumptions based on the Goldman-Hodgkin-Katz current equation^1,2^, is given by:

$I_{S}=P_{S}VF^{2}Z_{S}^{2}\frac{C_{S_{in}}-C_{S_{out}}e^{-VF\frac{Z_{S}}{RT}}}{RT\left( 1-e^{-VF\frac{Z_{S}}{RT}} \right)}$ (S2)

$I_{S}$ (A m^-2^) is the current density carried by ions *s* across a charge-selective droplet, $P_{S}$ (m s^-1^) is the permeability of *s* through the droplet, $V$ (V) is the generated voltage across the droplet, and $C_{S_{in}}$ and $C_{S_{out}}$ (mol m^-3^) are the concentrations of *s* inside and outside the droplet. For example, when formulated to consider only KCl in the high- and low-salt droplets, the total ionic current density $I$ (A m^-2^) is the sum of $I_{K}$ and $I_{Cl}$.

$I=\frac{VF^{2}}{RT\left( 1-e^{\frac{-VF}{RT}} \right)}\left[ \left( P_{K^{+}}C_{K_{in}^{+}}+P_{{Cl}^{-}}C_{{Cl}_{out}^{-}} \right)-\left( P_{K^{+}}C_{K_{out}^{+}}+P_{{Cl}^{-}}C_{{Cl}_{in}^{-}} \right)e^{\frac{-VF}{RT}} \right]$ (S3)

**1.2 Part 2**

Equations S1 to S3 formulate the relationship between the intrinsic electromotive force ($V_{Source}$, overall $V$ across cation-selective and anion-selective droplets of the power source) and the ideal ion flux of the droplet power source in electrochemical theory; in actual use and measurement, we also need to consider the ion flux in the low-salt droplet (Schematic S1a). In the low-salt droplet, ions of the salt initially present and inflowing oppositely-charged ions would move to neutralise the inflowing ions to keep electroneutrality, thus completing an ionic pathway. For example, inflowing Cl^-^ from right high-salt droplet and initial Cl^-^ in the low-salt droplet would move to neutralise the inflowing K^+^ ions from the left high-salt droplet. As a result, the ion fluxes from left and right of a power source unit will be equal to maintain electroneutrality. The voltage division can be modeled in terms of the internal resistance ($R_{Source}$) of the droplet power source (Schematic S1b). The resistance of each droplet is strongly dependent on the ionic strength within it^3,4^, so that the low-salt droplet contributes the main resistance in the system ($R_{Source}\approx R_{Low-salinity}$)^2,5^. At open circuit, the power source connects to a very large external resistance within the connected meter ($R_{Meter}\to\infty\gg R_{Source}$), forcing the droplet power source into a regime with high output voltage but very low current ($I=0$). Equation S3 then reduces to

${{V_{Meter}=V}_{Source}=V}_{OC}=\frac{RT}{F}\ln\left( \frac{P_{K^{+}}C_{K_{out}^{+}}+P_{{Cl}^{-}}C_{{Cl}_{in}^{-}}}{P_{K^{+}}C_{K_{in}^{+}}+P_{{Cl}^{-}}C_{{Cl}_{out}^{-}}} \right)$ (S4)

At short circuit ($R_{Meter}\to0 \ll R_{Source},V_{Meter}=0$), Equation S3 then reduces to

$I_{Meter}=I_{SC}=\frac{V_{Source}}{R_{Source}}\approx\frac{V_{OC}}{R_{Low-salinity}}$ (S5)

Therefore, the low-salt droplet affects $V_{OC}$ by its concentration gradient with the high-salt droplet (Extended Data Fig. 4a), and limits $I_{SC}$ by its resistance (Extended Data Fig. 4b and c). Decreasing salt concentration in the low-salt droplet will increase the $V_{OC}$ but decrease the $I_{SC}$ due to the increased resistance. As a trade-off, we used 0.01 M salt in the low-salt droplet except where noted.

**1.3 Part 3**

The ion flux of the droplet power source can be converted into electron flow in the external circuit by using chemically active electrodes (Schematic S1a). We used Ag/AgCl wire electrodes to contact with the first and the last high-salt droplets: on the anode side (droplet of high-salt connected to cation-selective droplet), anions are converted into electrons by Ag(solid) + Cl^-^ → AgCl(solid) + e^-^; on the cathode side (droplet of high-salt connected to anion-selective droplet), electrons are converted into anions by AgCl(solid) + e^-^ → Ag(solid) + Cl^-^. Therefore, as a whole, the droplet power source follows the law of electroneutrality.

**1.4 Part 4**

If neurons are embedded in the low-salt droplets, the inflowing ions will move in the extracellular space of the neurons and modulate neuronal activity. The modulation is the result of the ionic current^6-9^ generated by the droplet device. Hence, this modulation is dynamic and stops when the droplet device reaches the final balance of salt concentration.


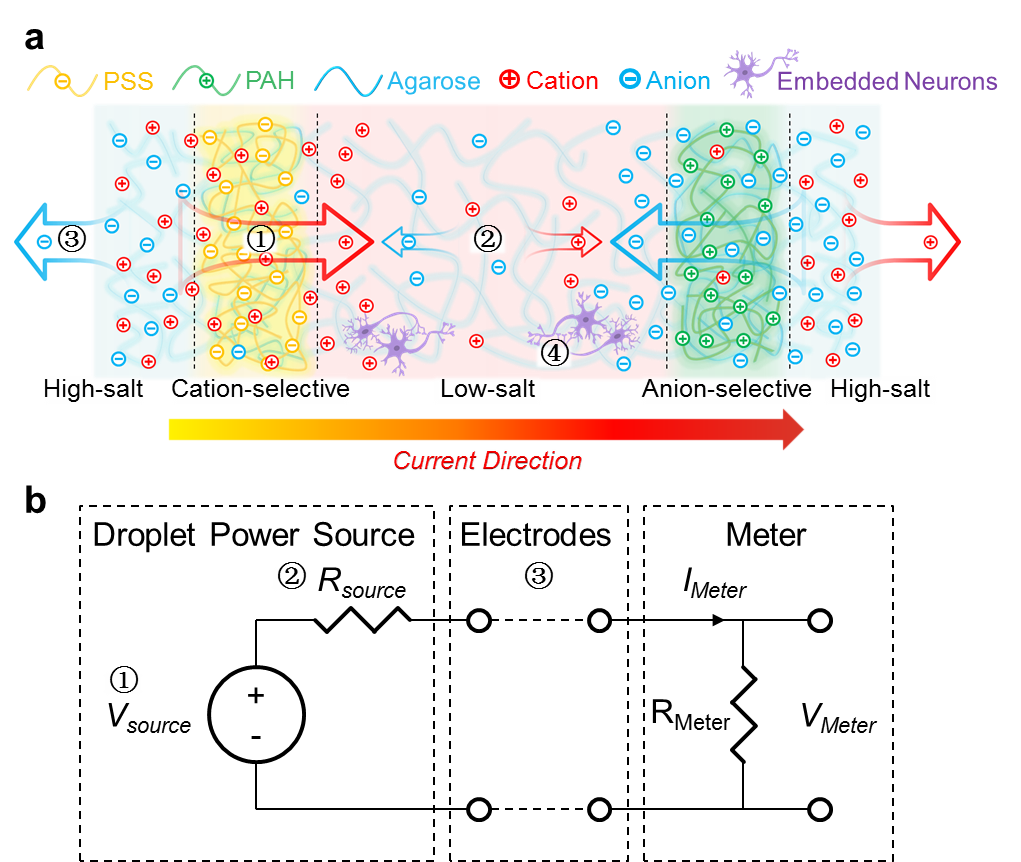


**Schematic S1 Schematic illustrations of the basic electrochemical and electrical mechanisms of the droplet power source and a measurement circuit. a**, The ion fluxes inside a droplet power source can be simplified into four components. (1) The ionic gradient between a high-salt and a low-salt droplet gives rise to an electromotive force and an ion flux across the charge-selective droplet; (2) inflowing ions (blue and red arrows) move at rates that maintain electroneutrality; (3) ions in the terminal high-salt droplets react at the electrodes to convert the ion flux into electron flow in an external circuit; (4) the internal ionic current affects the activities of embedded neurons. **b**, The equivalent electric circuit of the droplet power source, connected electrodes, and the measuring meter, corresponding to parts (1), (2), and (3) in (**a**).

**Supplementary Note 2. Research background**

Inspired by the electric eel, a soft hydrogel power source has been developed by Prof. Michael Mayer’s group as an alternative to conventional solid batteries^2,5^. Their system used ionic gradients between polyacrylamide hydrogels bounded by cation- and anion-selective hydrogel membranes to generate output electricity. However, their soft power source focused on large-area gel fabrication in order to increase the output voltage and power, while the potential material toxicity and assembly methods were not designed for microscale-to-mesoscale biological applications.

Firstly, they used acrylamide to build the gel scaffold of their power source by photoinitiated radical crosslinking to form polyacrylamide. However, acrylamide monomer has neurotoxic effects^10,11^ if it has not been fully reacted or washed out. Further, their cation- and anion-selective materials (3-sulfopropyl acrylate (potassium salt), 2-acrylamido-2-methylpropane sulfonic acid, and (3-acrylamidopropyl) trimethylammonium chloride) also have potential biocompatibility issues if they have not been fully crosslinked with the gel scaffold. Moreover, Mayer and colleagues used a scalable stacking or folding geometry, e.g., *Miura-ori* folding, to achieve mechanical contact of multiple power units in series while circumventing power dissipation before contact. However, this method is only suitable for large-area assembly and presents challenges in terms of methods for microscale assembly that avoid self-discharge before usage.

Notably, three- and six-droplet power sources have previously been constructed, based on the concentration cell^12^. These networks function by incorporating an engineered anion-selective *Staphylococcal* α-hemolysin nanopore into a lipid bilayer separating two droplets containing different concentrations of NaCl, thus converting the ionic gradient energy into output electricity. However, at this point, such a droplet power source is still in its infancy and faces a multitude of fundamental challenges that limit its applicability for powering useful activities. First, the use of a protein nanopore as the ion-selective material greatly limited the output current to ~60 pA, which is insufficient to drive most devices. Moreover, because the power source relied on a restricted salt concentration gradient (10-fold at a maximum) to prevent droplet coalescence, it ran for only less than an hour with a low open-circuit voltage, before ion and water transport across the bilayer, the latter by osmosis, balanced the salt concentrations and severely reduced the output. In addition, issues such as recharging capability, large-scale fabrication, remote triggering (stimulus-responsiveness), and energy generation in a biological environment remain unexplored in this context.

In brief, although previous work has presented inspiring examples of soft power sources, our research addresses previous limitations and demonstrates unique biological applications by the modulation of neuronal activity.

**Supplementary Note 3. Additional output optimization**

Based on the aforementioned discussion and previous work^1,2^, the electrogenic performance of the droplet power source depends on the following factors: the concentration gradient between the high-salt and low-salt droplets, the volume of each droplet, the ion permeabilities of the two charge-selective droplets, and the external resistance. Some of these factors are analyzed in the main text, while others are discussed here.

**3.1 Volume and internal resistance of droplets**

The volume of a droplet can range from femtoliters to microliters, which affects the output performance (Fig. 2a and b). For example, the internal resistance of the droplet power source depends on droplet volume. The low-salt compartment contains only 0.01 M salt and thus contributes most of the system’s electrical resistance ($R$, also see 1.2 Part 2), which is directly proportional to the length of the ionically conductive pathway ($l$) and inversely proportional to the average cross-sectional area ($S_{average}$) of the droplet (Equation S6). $\rho$ is the resistivity of the droplet. Relating $l$ and $S_{average}$ to the diameter ($D$) of the spherical droplet, we obtain:.

$R\approx\frac{\rho l}{S_{average}}=\frac{\rho D}{\frac{\frac{1}{6}\pi D^{3}}{D}}=\frac{6\rho}{\pi D}$ (S6)

Therefore, as the volume and hence the diameter decrease, the internal resistance of droplet power source will increase and the output performance will decrease.

**3.2 Ion permeabilities of the two charge-selective droplets**

According to the Donnan effect and the Goldman-Hodgkin-Katz equation^1,2,13^, an increased ratio of the permeability of the selected ion relative to the permeability of the disfavored ion across a charge-selective membrane results in an increased voltage across that membrane. Hence, it is important to choose charge-selective materials that have high permselectivities for counter ions. In our work, we used poly(sodium 4-styrenesulfonate) (PSS) and poly(allylamine hydrochloride) (PAH) as the cation-selective and anion-selective materials, respectively. Notably, PSS and PAH have been commonly used as ion exchange polymers for water treatment due to their biocompatibility and high permselectivity (> 10^3^)^14,15^. In addition, both PSS and PAH are water-soluble and can be mixed with agarose hydrogel on account of their good hydrophilicity and long-chain entanglement^15^. Therefore, we used these charge-selective materials to produce the high output performance of our droplet power source.

**3.3 External resistance**

The last influencing factor is the external resistance. The importance of impedance matching is well known for traditional galvanic cells, which have maximum output power ($P_{max}$) when the internal resistance ($R_{internal}$) equals the external resistance ($R_{load}$)^1^ (Equation S7). In previously reported work, the low-salt gel contributed most of the total internal resistance of a power unit^2,5^. Here, we constructed the resistance–voltage–power curves (Extended Data Fig. 4c) and obtained the maximum output power by connecting a series of known-load resistances to the power sources while monitoring the voltage across the load. By repeating this measurement with various sizes of droplets, we obtained the power densities ($\rho_{density}$) of power units with various droplet volumes (Fig. 2b). The total volume of a droplet power unit is five times of the volume of a single droplet. Shrinking down the size of droplets can slightly increase the internal resistance and thus decrease the output voltage/current (Equation S6); however, the increase of energy density is more significant (Equation S8). For example, by reducing the size to below 100 nanolitres (1.87 nL), the energy density of our droplet power source increased by approximately 100 times to 1.3 kW m^-3^.

$P_{max}=\frac{{V_{load}}^{2}}{R_{load}}=\frac{{V_{OC}}^{2}}{{4R}_{internal}}$ (S7)

$\rho_{density}=\frac{P_{max}}{5\times Volume}=\frac{6P_{max}}{5\pi D^{3}}$ (S8)

$D$ is the diameter of the droplet.

**Supplementary Note 4. Mechanisms of neuronal imaging**

Ca^2+^ serves as an intracellular second messenger that controls key functions that are necessary for many neuronal processes, including firing, synaptic plasticity, and gene transcription^16,17^. Hence, imaging Ca^2+^ in neurons is particularly important and has been commonly achieved by using fluorogenic dyes^18,19^. Fluorogenic Ca^2+^ indicators are widely used for in-cell measurement of agonist-stimulated and antagonist-inhibited Ca^2+^ signaling through G protein-coupled receptors, a large and active target class involved in neuronal activity. To test the activity of the neurons in our printed droplets, we performed Ca^2+^ imaging with Fluo-4 Direct™.

A Fluo-4 Direct™ calcium assay kit (Invitrogen, F10471) was used according to the manufacturer’s instructions. The assay kit can be used in the presence of complete culture media and will efficiently suppress background fluorescence without sacrificing the specific intracellular fluorescence generated in the assay. The visible excitation wavelength, high sensitivity, and large fluorescence increase upon binding Ca^2+^ have made Fluo-4 Direct™ the choice of indicator for our work. Importantly, contributions to baseline fluorescence by ions (e.g., K^+^, Cl^-^, or Ca^2+^) from the droplet devices are eliminated by the addition of a suppression dye (contained in the Fluo-4 Direct™). Therefore, the imaging reflects intracellular neuronal Ca^2+^ levels.

**Supplementary Note 5. Intracellular Ca^2+^ waves induced by ionic current**

We observed two differences between modulation induced by electrodes (Extended Data Fig. 6) and by the droplet devices. The response time and the relative fluorescence change of the induced Ca^2+^ wave were slightly longer (over 1 min) and lower respectively for the ionic current produced by using Ag/AgCl electrodes in Ca^2+^-free droplets, in agreement with previous studies on neuronal networks^20^. These results might be explained by a neuronal model of regenerative Ca^2+^ release and wave propagation^7,9,21,22^. In brief, Ca^2+^ in neurons is contributed from two main sources: external Ca^2+^ entry through voltage-gated calcium channels and internal Ca^2+^ release through channels in the endoplasmic reticulum (e.g., inositol trisphosphate receptors). Ca^2+^ waves can propagate and be amplified like the ‘toppling dominos’ when there is Ca^2+^ available by extracellular entry and/or by intracellular release (Schematic S2). Therefore, when we used the droplet devices with Ca^2+^ ions, the device might have induced both intracellular Ca^2+^ release and extracellular Ca^2+^ entry which caused the Ca^2+^ waves to be slightly faster (less than 15 s) and stronger compared to Ca^2+^-free electrical modulation. These results uncouple the electrical and ionic effects induced by the droplet devices on neurons. They also demonstrate the combination of electrical and ionic effects in one device, which cannot be achieved with conventional electrode-based stimulation.


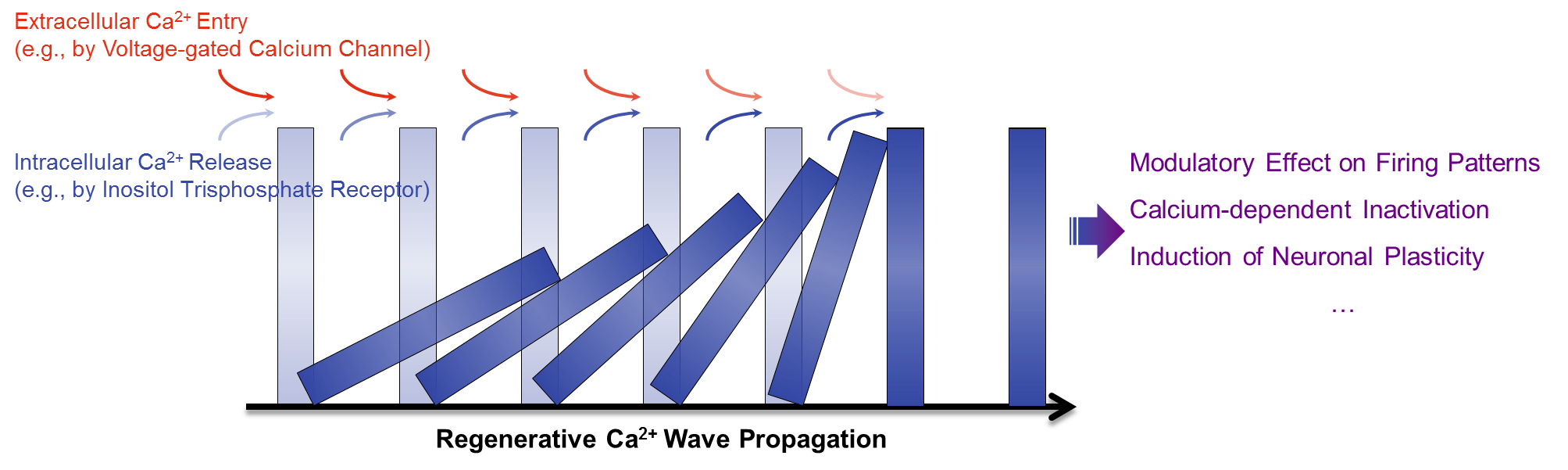


**Schematic S2 Model of regenerative Ca^2+^ release and wave propagation**^21^.
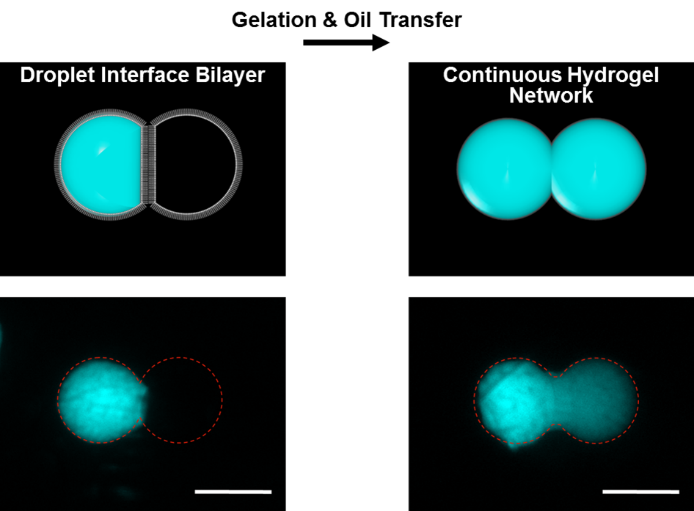


**Supplementary Fig. 1** **Rupture of the lipid bilayer between two pre-gel droplets.** After gelation and oil transfer, a continuous hydrogel is formed. Schematic (top) and fluorescence microscopy images (bottom) of a droplet pair before and after formation of the continuous hydrogel. The droplet pair was formed from an agarose pre-gel droplet and an agarose pre-gel droplet that contained the bilayer-impermeable dye ATTO-488 (1 μM, Sigma-Aldrich). Following gelation and oil transfer with lipid-free oil, the dye diffused into the adjacent droplet, indicating bilayer rupture. Scale bars, 250 μm.


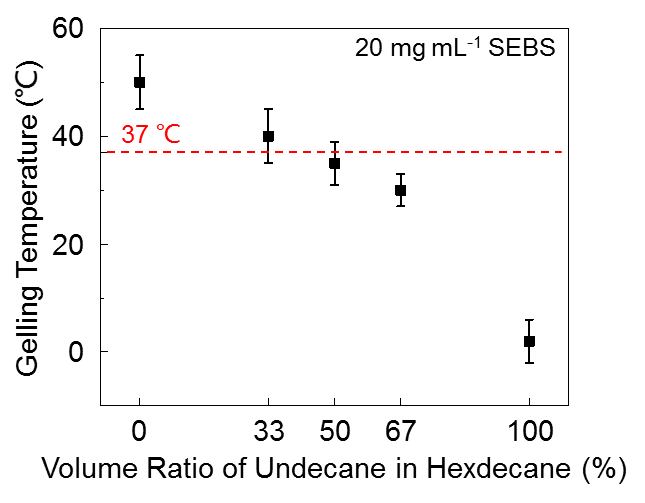


**Supplementary Fig. 2 The gelling temperature of the SEBS organogel.** We used undecane/hexadecane (50% v/v) for the oil transfer process, achieving a gelling temperature below 37 °C. The encapsulation was conducted by replacing the silicone oil with the molten organogel at the last oil transfer step.


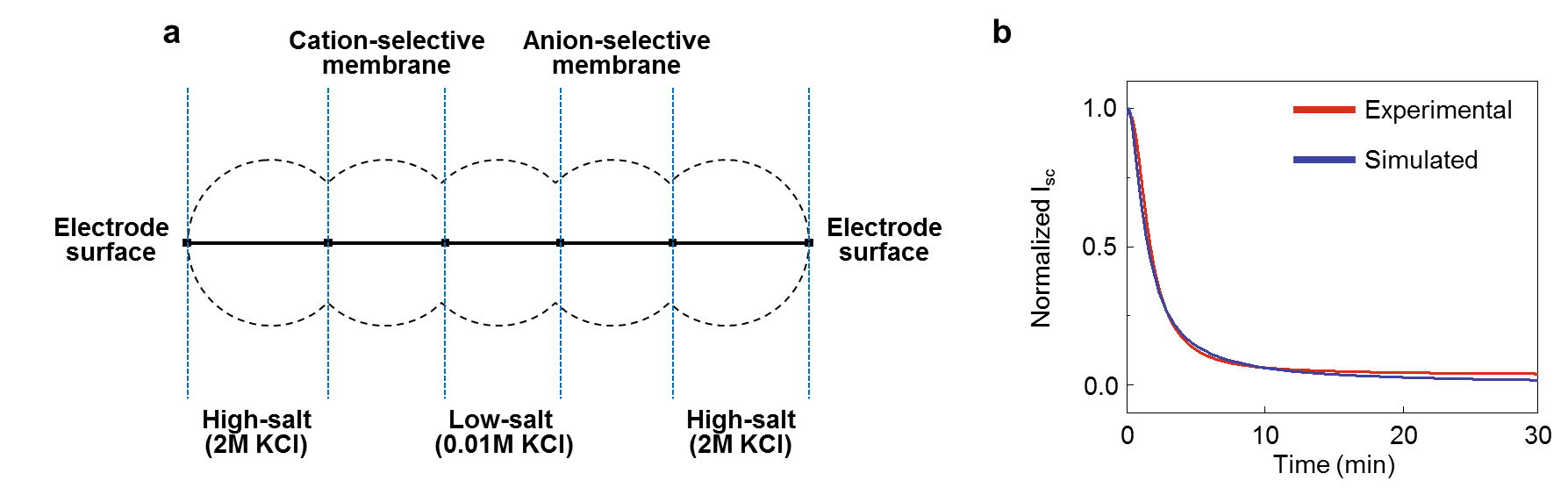


**Supplementary Fig. 3 Simulation of I_SC_ to support the experimental results.** **a**, The simulation layout with a droplet radius of 258 μm, corresponding to 50 nL in volume. **b**, Comparison of simulated and experimental results.


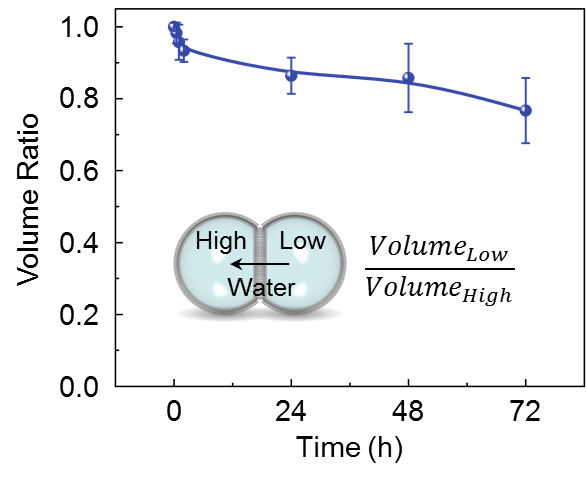


**Supplementary Fig. 4 Variation of the volume ratio over time.** The hydrogel droplet diameters were measured from photographs, and the droplet volumes were calculated with the assumption of spherical geometry. Volume ratio was with respect to the initial volume values (0 h) of each experiment. Data are presented as mean ± s.d. (n = 3).


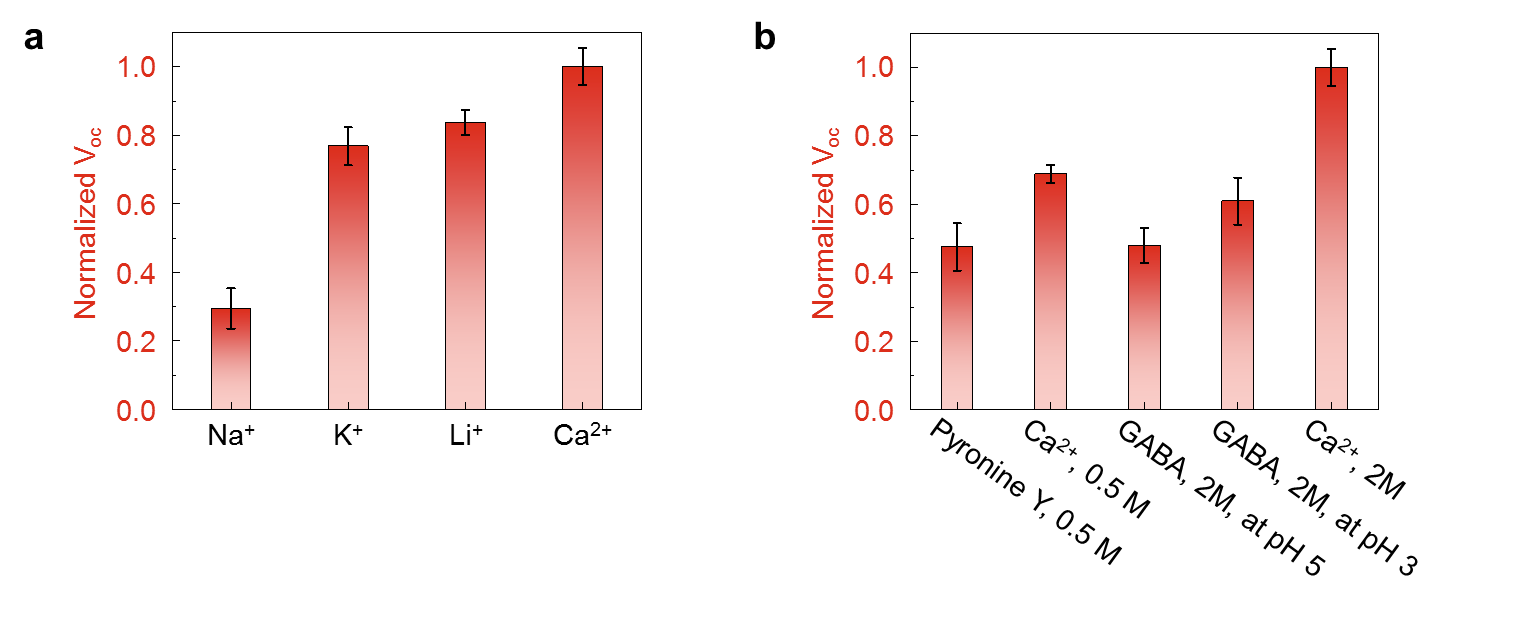


**Supplementary Fig. 5 Influence of the cations in the high- and low-salt droplets on electrical output**. Na^+^, K^+^, Ca^+^ and the cationic forms of pyronine Y and GABA were examined, all as chloride salts. **a**, Divalent Ca^2+^ ions carry more charge than the monovalent ions and therefore produce a higher voltage under the same concentration gradient (200-fold). The combined effects of a higher aqueous ionic mobility^14^ (K^+^: 7.62×10^-8^ m^2^ s^-1^ V^-1^, Na^+^: 5.19×10^-8^ m^2^ s^-1^ V^-1^) and a higher affinity for the cation-selective polystyrene sulfonate chain^23^ (K^+^: 2.27, Na^+^: 1.58) produce a higher output voltage for K^+^. **b**, Comparison with Ca^2+^ ions of the V_OC_ values of the cationic forms of pyronine Y and GABA at the concentrations shown.


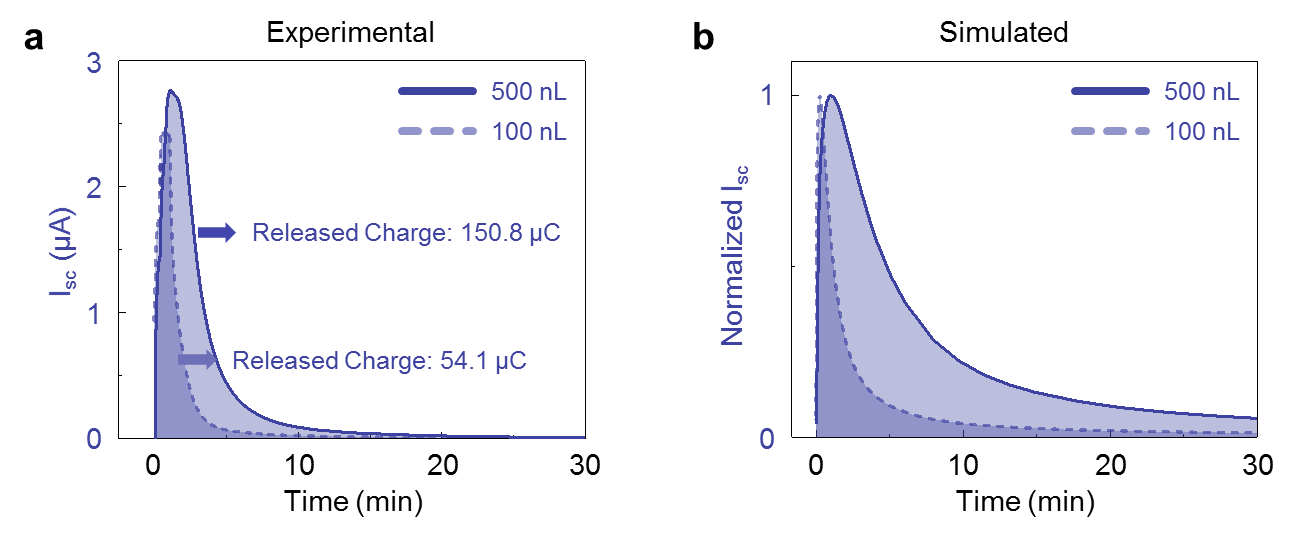


**Supplementary Fig. 6 I_SC_ versus time for two droplet power units with different volumes.** We integrated the current traces over time to calculate the capacity of the droplet power sources during each discharge. **a**, Experimental results. **b**, Simulations based on the same structural setup as the experimental droplet power sources (Supplementary Fig. 3).


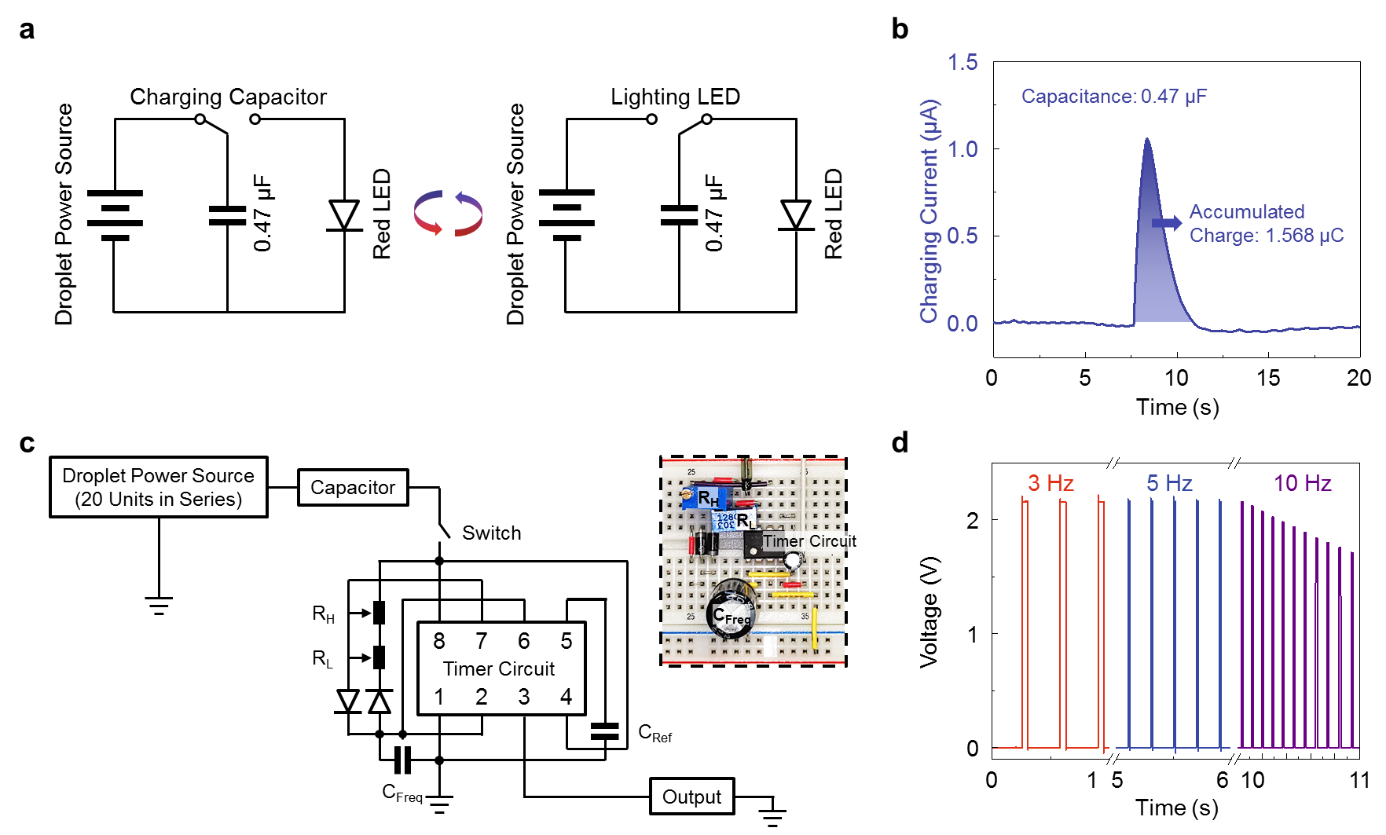


**Supplementary Fig. 7 Charging of electronic components and circuits. a**, Charging a capacitor and lighting an LED with the droplet power source. **b**, The charging current of a 0.47 μF capacitor. The blue area shows the integration area of the current trace over time, indicating the accumulated charge in the capacitor (~1.57 μC) during one charging cycle. **c**, Charging a pulse generator circuit with the droplet power source. The pulse generator circuit was connected with the droplet power source using a capacitor for charge collection. Inset, a photograph of the pulse generator circuit based on a 555-timer chip. **d**, By tuning the value of R_H_ and R_L_, the pulse frequency was tuned from 3 to 10 Hz. Higher frequencies led to the faster energy consumption of the droplet power source.


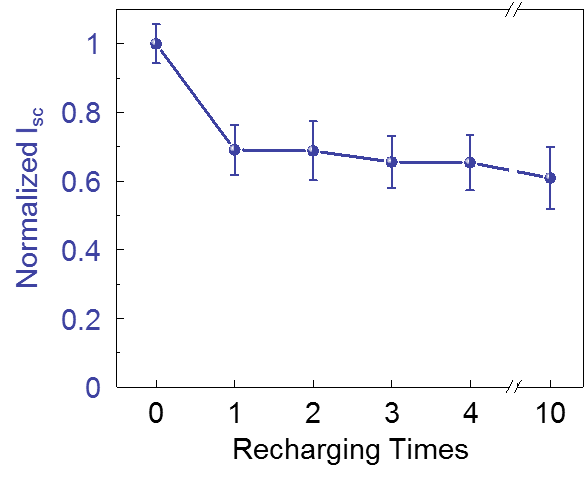


**Supplementary Fig. 8 Recharging a droplet power source network.** A droplet power source network (Fig. 3e and h) was recharged with a reversed 200 mV voltage for 5 min. I_SC_ was recorded and the cycle was repeated 10 times. Data are presented as mean ± s.d. (n = 5).


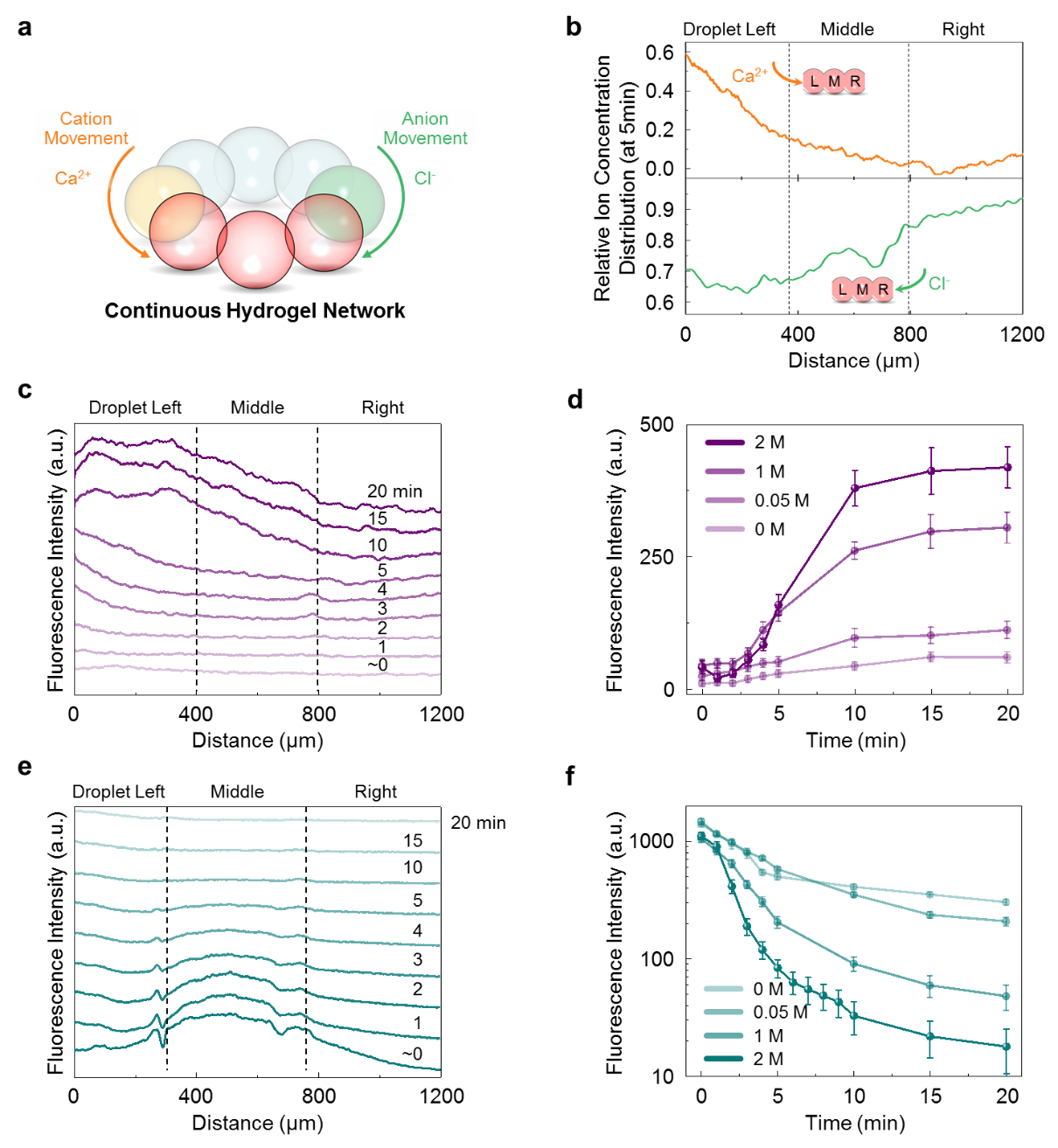


**Supplementary Fig. 9 Fluorescence monitoring of ion fluxes from droplet devices. a**, In the front three low-salt droplets (red), inflowing cations will move from left to right and inflowing anions right to left, creating an ionic current in the three droplets. **b** Relative concentration distributions of Ca^2+^ (orange) and Cl^-^ (green) across the three low-salt droplets, calculated from line scans of ﬂuorogenic Ca^2+^ and Cl^-^ indicators at 5 min (Methods). The volume of each droplet was ~500 nL. **c**, Line scans of the fluorescence signals across the three low-salt droplets (red in '**a**'), showing the Ca^2+^ concentration with time. **d**, The salt concentration in the high-salt droplets affects the speed and intensity of the cation flux. Data were from the front left droplet. **e**, Line scans of the fluorescence signals across the three low-salt droplets, showing the Cl^-^ concentration with time. Decreased fluorescence indicates increased Cl^-^. **f**, The salt concentration in the high-salt droplets affects the speed and intensity of the anion flux. Data were from the front right droplet. All data are presented as mean ± s.d. (n = 3).


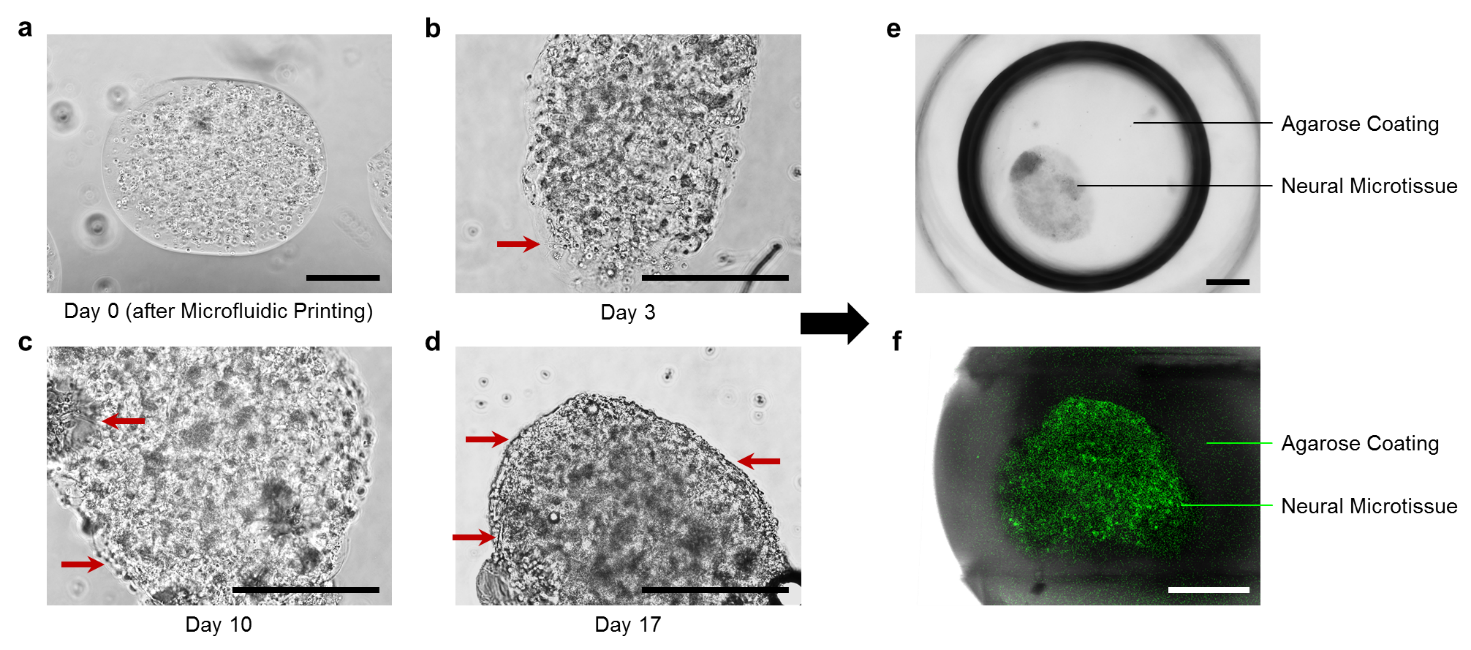


**Supplementary Fig. 10 3D neural microtissues in culture. a**–**d**, Microscopy images of neural microtissues at: **a**, day 0 (right after generation by a microfluidic system); **b**, day 3; **c**, day 10; **d**, day 17. The red arrows mark stretched and entangled neural processes, which indicate the formation of neuronal connections. **e** and **f**, Bright-field microscopy (**e**) and overlaid confocal (**f**) image of agarose droplets that contained neural microtissues at day 10. Neurons were stained with Calcein-AM. Scale bars, 250 μm.

**
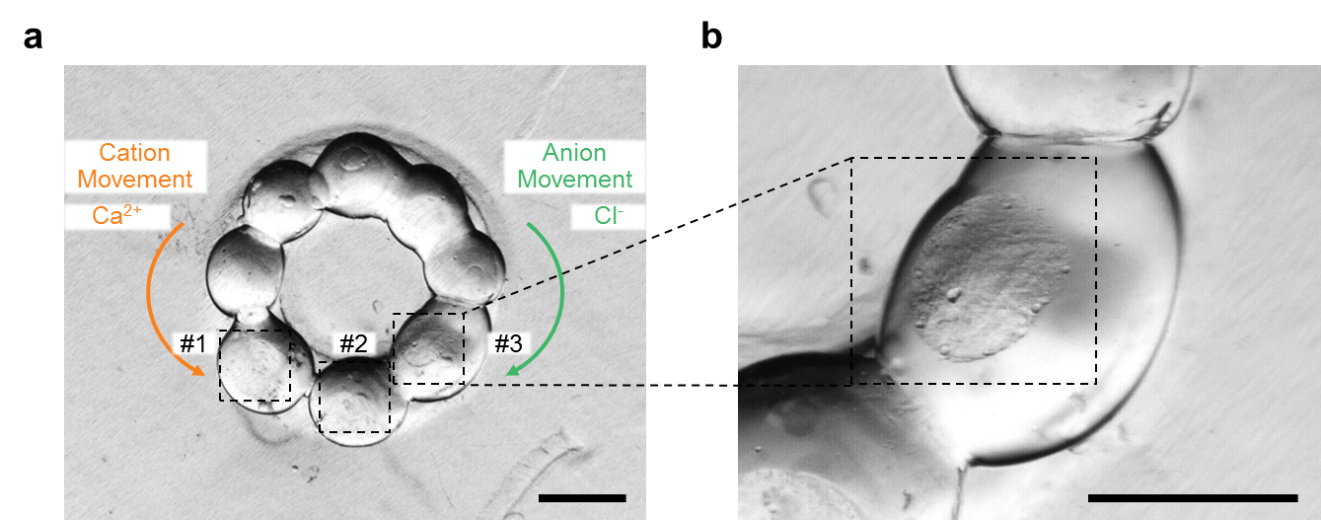
**

**Supplementary Fig. 11 Images of the droplet ring for triggering neuronal modulation. a**, A droplet device attached to droplets containing neural microtissues (three bottom droplets, #1, #2, and #3). **b**, A zoom-in view of droplet #3, which contains a neural microtissue at day 3. Scale bars, 500 μm.


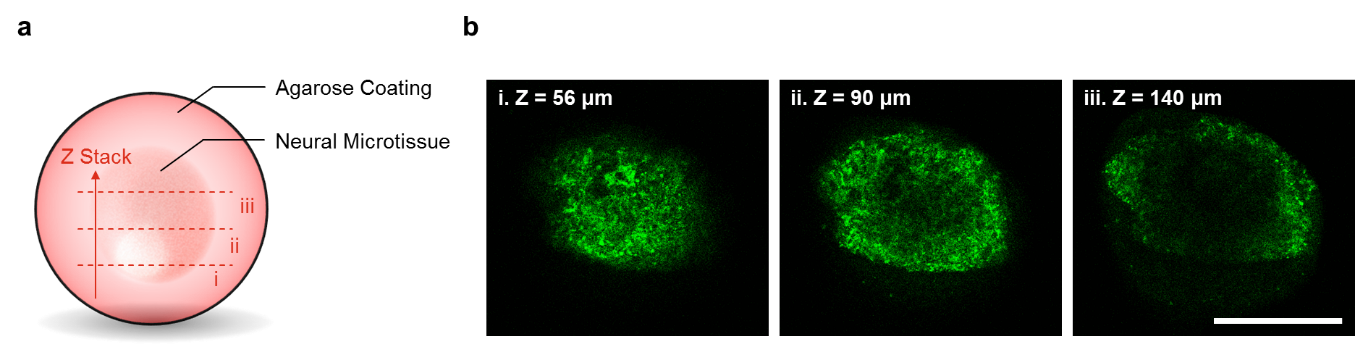


**Supplementary Fig. 12 XYZ scanning of a neuron-containing droplet (day 17) by confocal microscopy. a**, Schematic of the three Z stacks. **b**, Dark-field images. The bottom of the embedded neural microtissue was Z = 0 μm. Neurons were stained with Fluo-4 Direct™. Scale bar, 300 μm. Due to limited light-penetration, deeper stacks (e.g., Z = 140 μm) could only be imaged around the boundary.

**
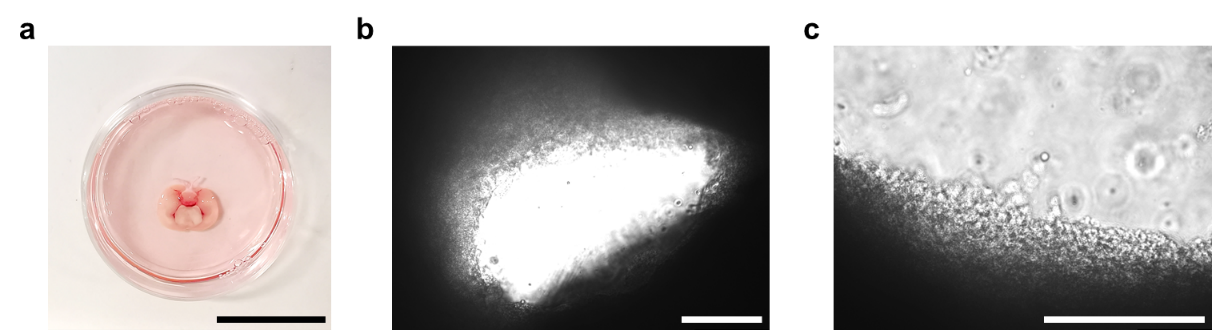
**

**Supplementary Fig. 13 Images of mouse brain slices. a**, A mouse brain slice in culture medium. Scale bar, 1.5 cm. **b**, Using a 1 mm biopsy punch (KAI), part of the brain slice was cut out and embedded in a droplet by coating with agarose (Methods). **c**, Bright-field image of the boundary of a brain slice, where neurons are visible. Scale bars in (**b**) and (**c**), 65 μm.

**
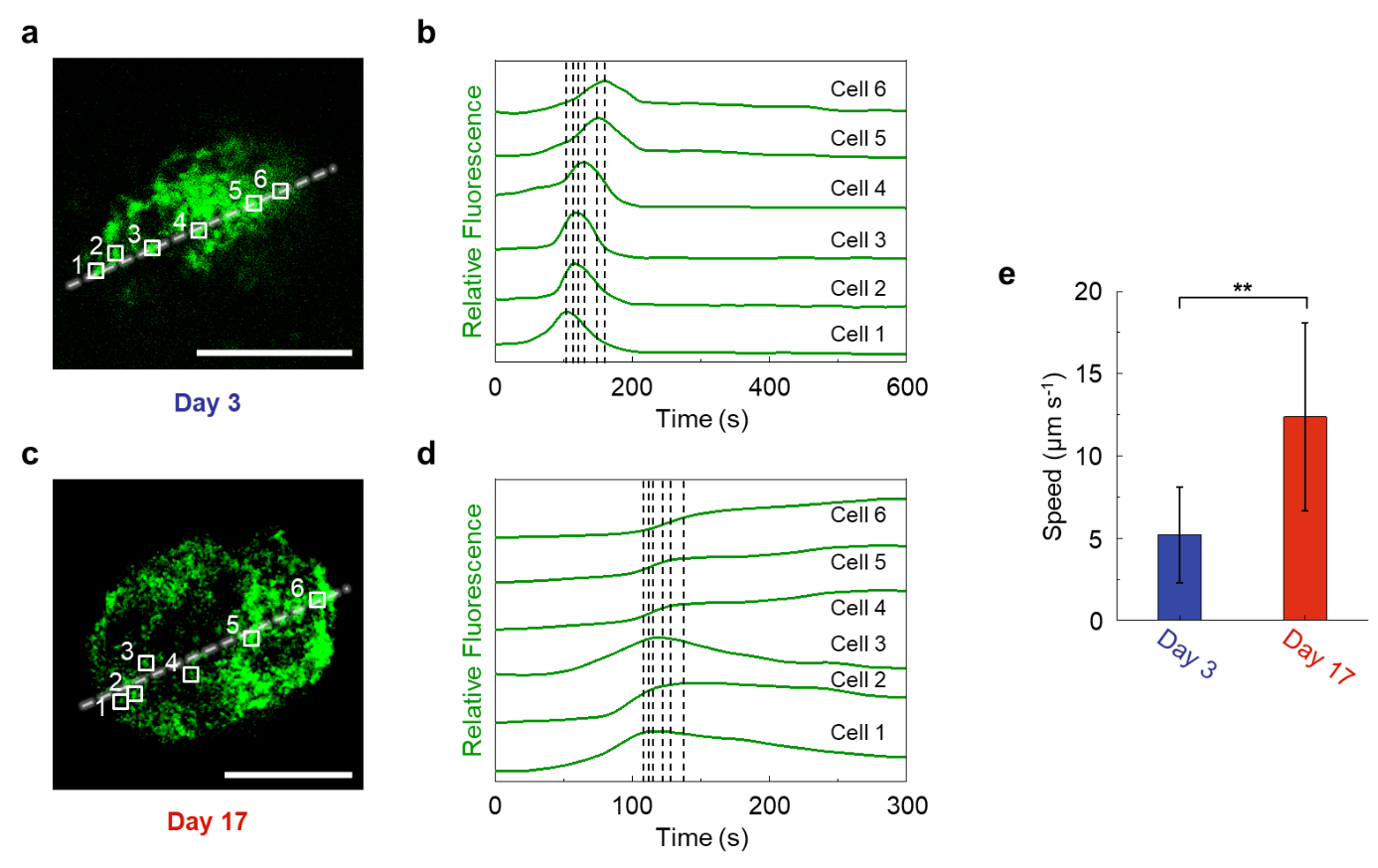
**

**Supplementary Fig. 14 Calculation of the** **Ca^2+^ wave** **propagation speed from the fluorescence intensity of individual neurons. a**, Selected neurons in a day 3 neural microtissue along the direction of the Ca^2+^ wave (white dashed line). **b**, Time-dependent changes of intracellular Ca^2+^ concentration in cells marked 1–6 in (**a**). **c** and **d**, Selected neurons in a day 17 neural microtissue (**c**) and their changes in intracellular Ca^2+^ concentration (**d**). **e**, Speeds of Ca^2+^ wave propagation at day 3 and day 17. We picked 6 neurons in each neural microtissue (5 data values), and 3 neural microtissues with the same culture times for analysis (n = 15). Data are presented as mean ± s.d. (**denotes P < 0.01, two-sample t-test). Scale bars, 150 μm.

**Reference**

1. Xu, J., Sigworth, F. J. & LaVan, D. A. Synthetic protocells to mimic and test cell function. *Adv. Mater.* **22**, 120-127 (2010).

2. Schroeder, T. B. H. *et al.* An electric-eel-inspired soft power source from stacked hydrogels. *Nature* **552**, 214-218 (2017).

3. Długołęcki, P. *et al.* On the resistances of membrane, diffusion boundary layer and double layer in ion exchange membrane transport. *J. Membr. Sci.* **349**, 369-379 (2010).

4. Galama, A. *et al.* Membrane resistance: The effect of salinity gradients over a cation exchange membrane. *J. Membr. Sci.* **467**, 279-291 (2014).

5. Guha, A. *et al.* Powering Electronic Devices from Salt Gradients in AA-Battery-Sized Stacks of Hydrogel-Infused Paper. *Adv. Mater.* **33**, 2101757 (2021).

6. Wang, C. *et al.* Inverted battery design as ion generator for interfacing with biosystems. *Nat. Commun.* **8**, 1-7 (2017).

7. Ryglewski, S., Pflueger, H. J. & Duch, C. Expanding the Neuron's Calcium Signaling Repertoire: Intracellular Calcium Release via Voltage-Induced PLC and IP3R Activation. *PLoS Biol.* **5**, e66 (2007).

8. Zhang, L. I. & Poo, M.-m. Electrical activity and development of neural circuits. *Nat. Neurosci.* **4**, 1207-1214 (2001).

9. Scemes, E. & Giaume, C. Astrocyte calcium waves: what they are and what they do. *Glia* **54**, 716-725 (2006).

10. Spencer, P. S. & Schaumburg, H. H. Nervous system degeneration produced by acrylamide monomer. *Environ. Health Perspect.* **11**, 129-133 (1975).

11. Hamilton, P. D., Aliyar, H. & Ravi, N. Biocompatibility of novel polyacrylamide copolymer suitable for intra–ocular lenses. *Invest. Ophthalmol. Visual Sci.* **45**, 1728-1728 (2004).

12. Holden, M. A., Needham, D. & Bayley, H. Functional bionetworks from nanoliter water droplets. *J. Am. Chem. Soc.* **129**, 8650-8655 (2007).

13. Crow, D. R. *Principles and applications of electrochemistry*. Vol. 6. Electrode potentials and electrochemistry cells (Routledge, 2017).

14. Zuo, K. *et al.* Selective membranes in water and wastewater treatment: Role of advanced materials. *Mater. Today* **50**, 516-532 (2021).

15. White, N., Misovich, M., Yaroshchuk, A. & Bruening, M. L. Coating of Nafion Membranes with Polyelectrolyte Multilayers to Achieve High Monovalent/Divalent Cation Electrodialysis Selectivities. *ACS Appl. Mater. Interfaces* **7**, 6620-6628 (2015).

16. Zhou, L. *et al.* Lipid-bilayer-supported 3D printing of human cerebral cortex cells reveals developmental interactions. *Adv. Mater.* **32**, 2002183 (2020).

17. Citri, A. & Malenka, R. C. Synaptic plasticity: multiple forms, functions, and mechanisms. *Neuropsychopharmacol.* **33**, 18-41 (2008).

18. Grienberger, C. & Konnerth, A. Imaging calcium in neurons. *Neuron* **73**, 862-885 (2012).

19. Ricardo Augusto de Melo Reis, F., Hércules Rezende, Fernando Garcia de Mello. Cell Calcium Imaging as a Reliable Method to Study Neuron–Glial Circuits. *Front. Neurosci.* **14** (2020).

20. Fujii, Y., Maekawa, S. & Morita, M. Astrocyte calcium waves propagate proximally by gap junction and distally by extracellular diffusion of ATP released from volume-regulated anion channels. *Sci. Rep.* **7**, 13115 (2017).

21. Ross, W. N. Understanding calcium waves and sparks in central neurons. *Nat. Rev. Neurosci.* **13**, 157-168 (2012).

22. Warren, N. J., Tawhai, M. H. & Crampin, E. J. Mathematical modelling of calcium wave propagation in mammalian airway epithelium: evidence for regenerative ATP release. *Exp. Physiol.* **95**, 232-249 (2010).

23. Nakamura, T. *et al.* Risks and Benefits of Sodium Polystyrene Sulfonate for Hyperkalemia in Patients on Maintenance Hemodialysis. *Drugs R&D* **18**, 231-235 (2018).
